# Development of an Efficient C-to-T Base-Editing System and Its Application to Cellulase Transcription Factor Precise Engineering in Thermophilic Fungus *Myceliophthora thermophila*

**DOI:** 10.1128/spectrum.02321-21

**Published:** 2022-05-24

**Authors:** Chenyang Zhang, Nan Li, Lang Rao, Jingen Li, Qian Liu, Chaoguang Tian

**Affiliations:** a Key Laboratory of Systems Microbial Biotechnology, Tianjin Institute of Industrial Biotechnology, Chinese Academy of Sciences, Tianjin, People’s Republic of China; b National Technology Innovation Center of Synthetic Biology, Tianjin, People’s Republic of China; c University of Chinese Academy of Sciences, Beijing, People’s Republic of China; d College of Biotechnology, Tianjin University of Science & Technology, Tianjin, People’s Republic of China; Westerdijk Fungal Biodiversity Institute

**Keywords:** *Myceliophthora thermophila*, base editing, cytosine base editor, precise engineering, CRISPR/Cas9 system, MtCLR-2, cellulase

## Abstract

*Myceliophthora thermophila* is a thermophilic fungus with great potential in biorefineries and biotechnology. The base editor is an upgraded version of the clustered regularly interspaced short palindromic repeats (CRISPR)-dependent genome-editing tool that introduces precise point mutations without causing DNA double-strand breaks (DSBs) and has been used in various organisms but rarely in filamentous fungi, especially thermophilic filamentous fungi. Here, for the first time, we constructed three cytosine base editors (CBEs) in *M. thermophila*, namely, evolved apolipoprotein B mRNA-editing enzyme catalytic subunit 1 (APOBEC1) cytosine base editor 4 max (Mtevo-BE4max), bacteriophage Mu Gam protein cytosine base editor 4 max (MtGAM-BE4max), and evolved CDA1 deaminase cytosine base editor (Mtevo-CDA1), and efficiently inactivated genes by precisely converting three codons (CAA, CAG, and CGA) into stop codons without DSB formation. The Mtevo-CDA1 editor with up to 92.6% editing efficiency is a more suitable tool for cytosine base editing in thermophilic fungi. To investigate the function of each motif of the cellulase transcription factor *M. thermophila* CLR-2 (MtCLR-2), we used the Mtevo-CDA1 editor. The fungal-specific motif of MtCLR-2 was found to be strongly involved in cellulase secretion, conidium formation, hyphal branching, and colony formation. Mutation of the fungus-specific motif caused significant defects in these characteristics. Thus, we developed an efficient thermophilic fungus-compatible base-editing system that could also be used for genetic engineering in other relevant filamentous fungi.

**IMPORTANCE** A CRISPR/Cas-based base-editing approach has been developed to introduce point mutations without inducing double-strand breaks (DSBs) and attracted substantial academic and industrial interest. Our study developed the deaminase-cytosine base-editing system to efficiently edit three target genes, *amdS*, *cre-1*, and the essential cellulase regulator gene *Mtclr-2*, in *Myceliophthora thermophila*. A variety of point mutations in the target loci of the DNA-binding domain and fungus-specific motif of *M. thermophila* CLR-2 (MtCLR-2) were successfully generated via our base editor Mtevo-CDA1 to elucidate its function. Here, we show that the DNA-binding domain of MtCLR-2 is important for the fungal response to cellulose conditions, while its fungus-specific motif is involved in fungal growth. These findings indicate that our base editor can be an effective tool for elucidating the functions of motifs of target genes in filamentous fungi and for metabolic engineering in the field of synthetic biology.

## INTRODUCTION

Lignocellulosic biomass is an abundant, sustainable, renewable, and conventional energy source for the production of second-generation biofuels and biochemicals (1). In nature, saprobic ascomycete and basidiomycete filamentous fungi are the main decomposers of plant biomass (2). They decompose lignocellulose into easy-to-use energy by secreting large amounts of lignocellulosic enzymes (2). The thermophilic filamentous fungus *Myceliophthora thermophila* has a variety of industrial characteristics, such as high-temperature fermentation, the ability to secrete proteins with high efficiency, and the capacity to grow rapidly with a single cellulose carbon source (3–7), conferring great potential for development in bioindustrial applications. Recently, the gene-editing tool clustered regularly interspaced short palindromic repeats/CRISPR-associated (CRISPR/Cas) was developed by our group in *M. thermophila* and has accelerated research progress on gene function, genetic engineering, and metabolic engineering (8). However, there is still a need for more powerful and universal genome-editing tools to promote a wider range of fungal biotechnological applications.

The CRISPR/Cas-based gene-editing system is an efficient genetic modification tool for gene disruption by Cas nuclease, which can induce DNA double-strand breaks (DSBs) at a precise target location (9). DSBs can be repaired by endogenous repair mechanisms, including nonhomologous end joining (NHEJ) to mediate random insertions or deletions and homology-directed repair (HDR) to induce precise gene mutations using exogenous DNA templates (10, 11). The base editor (BE) is a new genome-editing system that creates precise single-nucleotide substitutions at genomic DNA targets without requiring DSBs, delivery of DNA donor templates, or relying on HDR and NHEJ (12). Because base editing does not induce DSBs, it can minimize the DNA injury created by DSBs and undesirable random mutation indels generated by NHEJ (13, 14).

Base editors consist of a catalytically inactive form of Cas9 (dcas9) or a Cas9 nickase mutant (nCas9), cytidine deaminases, such as apolipoprotein B mRNA-editing enzyme catalytic subunit 1 (APOBEC1) and activation-induced cytidine deaminase (AID), and a uracil glycosylase inhibitor (UG1) (14, 15). For example, third-generation base editor 3 (BE3) is a fusion protein composed of rat APOBEC1 (rAPOBEC1), a uracil glycosylase inhibitor (UGI), and the Cas9-D10A nickase mutant (14). The single guide RNA (sgRNA) consists of a 20-bp protospacer sequence that recognizes the target site by base pairing and a downstream sgRNA scaffold sequence (16). The target DNA contains the 20-bp target sequences followed by the requisite 3-bp protospacer adjacent motif (PAM) 5'-NGG (9). The mature sgRNA guides nCas9 to specific 20-nucleotide (nt) genomic loci, and nCas9 then introduces a single-strand break at the target DNA upstream of the PAM (14, 17, 18). Following BE3 binding to target sites mediated by sgRNA, rAPOBEC1 converts a targeted cytosine (C) into uracil (U), and UGI inhibits U removal by DNA glycosylases (14). The resulting G:U mismatch is then converted into an A:T base pair following Cas9-mediated nicking of the G-containing DNA strand followed by DNA synthesis (12). A variety of novel cytosine base editors (CBEs) have been developed in recent years, including cytosine base editor 4 (BE4) (19), bacteriophage Mu Gam protein-cytosine base editor 4 (GAM-BE4) (19), and optimizing APOBEC1-cytosine base editor 4 max (BE4max) (20). For instance, the fourth-generation base editor (BE4) is an upgraded version of BE3, which was constructed by extending the linker lengths in APOBEC1-nCas9 and nCas9-UGI and adding another copy of UGI (19). To further reduce indel formation during the base-editing process, the bacteriophage protein Gam was fused to the N terminus of BE4 to obtain GAM-BE4 (19). An upgraded version of BE4, BE4max, was subsequently generated by modifying the nuclear localization signal (NLS), optimizing codon usage, and reconstituting the deaminase component. The BE4max corrects pathogenic single-nucleotide polymorphisms (SNPs) in various types of mammalian cells and greatly improves efficiency compared with BE4 (20). Cytosine base editing can also use the cytidine deaminase 1 (CDA1) ortholog PmCDA1 from sea lamprey in base editor constructs to generate the fusion protein nCas9-CDA1-UGI (15). Subsequently evolved CBEs, such as evolved base editor 4 max (evo-BE4max) and evolved CDA1 cytosine base editor (evo-CDA1), have recently been developed to overcome the targeting sequence limitations of wild-type CBEs and enable the editing of cytosines in a GC environment with up to 26-fold higher efficiency than BE4 (21). Although base-editing tools are efficiently applied in mammalian, animal, and plant cells (14, 22–29), there is still a need to develop and improve base editors in filamentous fungi.

Gene-editing tools, such as the CRISPR/Cas9 system and base editors, are applied in genetic engineering, but the controversial issue of off-target effects has affected the application of this system (30–35). For example, the CRISPR/Cas9 system has been reported to produce large fragment deletions and complex chromosomal rearrangements at the targeted sites in mouse embryonic stem cells, mouse hematopoietic progenitors, and a human differentiated cell line (33). A genome-wide assessment of off-target effects by whole-genome and digenome sequencing found that the rAPOBEC1-nCas9 base editor was highly specific and induced cytosine-to-uracil conversions at only 18 ± 9 sites in the human genome for each sgRNA (34). Meanwhile, genome-wide off-target analysis in mouse embryos showed that cytosine base editing induced single-nucleotide variants (SNVs) at higher frequencies than CRISPR-Cas9 or adenine base editor (ABE) (36). One study similarly found in rice plants that the CBE system induced substantial genome-wide off-target mutations, which are mostly the C→T type of SNVs and appear to be enriched in genic regions, while the ABE system has good fidelity (37).

In this study, three cytosine base editors (CBEs; Mtevo-BE4max, MtGAM-BE4max, and Mtevo-CDA1) were developed in the thermophilic fungus *M. thermophila* and successfully applied for genomic editing. We found that the CBE system efficiently inactivates target genes through the induction of stop codons in the open reading frame (ORF) of the genes. This system relies on the ability to convert three codons (CAA, CAG, or CGA) into stop codons (TAA, TAG, or TGA). Among these three cytosine base editors, the Mtevo-CDA1 editor, with up to 92.6% editing efficiency, is a more suitable tool for cytosine base editing in *M. thermophila*. Recently, CLR-2 was identified as an essential transcription factor for cellulase expression and cellulose degradation in filamentous fungi, which was first identified in Neurospora crassa, and its orthologs were characterized in other fungi, including Aspergillus nidulans, Aspergillus niger, and Trichoderma reesei (38–43). However, the function of the ortholog of CLR-2 in *M. thermophila* (MtCLR-2) for regulating the production of cellulases remains less clearly defined. There are several fungus-specific functional domains in CLR-2, including a DNA-binding motif and fungus-specific motif, which are conserved in filamentous fungi. To investigate the function of each motif of MtCLR-2, we generated multiple strains with point mutations at the DNA-binding motif and the fungus-specific motif of MtCLR-2 using the Mtevo-CDA1 editor. Our results showed that MtCLR-2 is a key transcription factor involved in cellulase secretion, conidium formation, hyphal branching, and colony formation. Disruption of the MtCLR-2 fungus-specific motif led to significant defects in these features. Taking these findings together, we developed an efficient base-editing system in thermophilic fungi, which can also be applied for genetic engineering in other filamentous fungi.

## RESULTS

### Rational design of a cytosine base-editing system in *M. thermophila*.

**FIG 1:**
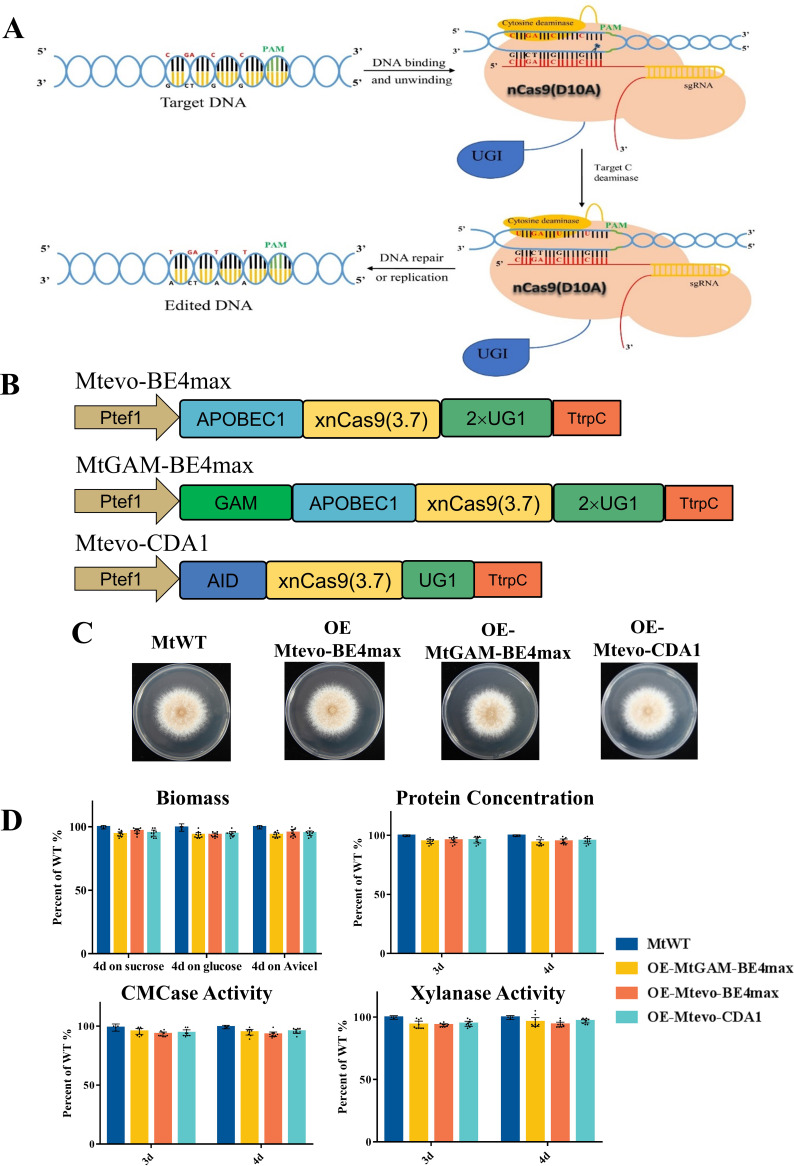
Schematic representation of the cytosine base-editing system in this study and phenotypic analysis of constitutive CBE-expressing strains. (A) Schematic diagram of the working principle of cytosine base editors. (B) Representation of the three cytosine base editors. P*tef1*, the *tef1* promoter; APOBEC1B, apolipoprotein B mRNA-editing enzyme catalytic subunit 1; AID, activation-induced cytidine deaminase; xnCas9 (3.7), Cas9-D10A nickase mutant; UGI, uracil glycosylase inhibitor; T*trpC*, T*trpC* terminators. (C) Colony growth and sporulation of OE-Mtevo-BE4max, OE-MtGAM-BE4max, OE-Mtevo-CDA1, and wild-type (MtWT) strains on minimal medium plates after 4 days of culture. (D) Mycelial biomass of OE-Mtevo-BE4max, OE-MtGAM-BE4max, OE-Mtevo-CDA1, and MtWT from cultures on sucrose, glucose, and Avicel after 4 days of culture. Assays for the protein concentrations of OE-Mtevo-BE4max, OE-MtGAM-BE4max, OE-Mtevo-CDA1, and MtWT in inducing medium with 2% Avicel after 3 and 4 days of culture. Each black dot represents an individual transformant. Bars marked by asterisks in each group differ significantly from the unmarked bars (Tukey’s honestly significant difference [HSD] test; *, *P < *0.05). Error bars indicate the standard deviation (SD) from multiple replicates.

To investigate the potential editing activity of base-editing systems in filamentous fungi, we designed and developed three nCas9 (D10A)-based genome-editing vectors: Mtevo-BE4max, MtGAM-BE4max, and Mtevo-CDA1 (Fig. 1A and B). Previously, we successfully harnessed the *tef1* promoter P*tef1* and U6 sgRNA promoter U6p to drive the expression of Cas9 and sgRNAs, respectively (8). Thus, in this study, the codon-optimized nCas9 (D10A) gene, the corresponding deaminase, uracil DNA glycosylase inhibitor (UGI), and the corresponding sgRNA were expressed under the control of P*tef1* and U6p, respectively. To test whether the constitutive expression of the three Mtevo-BE4max, MtGAM-BE4max, and Mtevo-CDA1 editors is harmful to the strain, we individually transformed each base editor in the *M. thermophila* wild-type strain (MtWT). After these three transformations, we selected positive transformants from each vector and named them OE-Mtevo-BE4max, OE-MtGAM-BE4max, and OE-Mtevo-CDA1, respectively (collectively referred to as OE-CBE strains). Colony growth, secreted protein production, enzyme activities, and mycelial biomass of the OE-CBE strains were examined in parallel with those of MtWT (Fig. 1C and D). These OE-CBE strains and MtWT exhibited similar colony growth when grown on a variety of carbon sources (Fig. 1C and Fig. S1 in the supplemental material). No significant differences in secreted protein and enzyme activities were observed after 3 or 4 days of fermentation on 2% Avicel liquid medium (Fig. 1D). Taken together, the results indicated that constitutive expression of the Mtevo-BE4max, MtGAM-BE4max, and Mtevo-CDA1 editors did not alter colony growth, sporulation, mycelium biomass, or cellulase production of *M. thermophila.*

### Cytosine base-editing system-directed base mutagenesis in *M. thermophila*.

**FIG 2:**
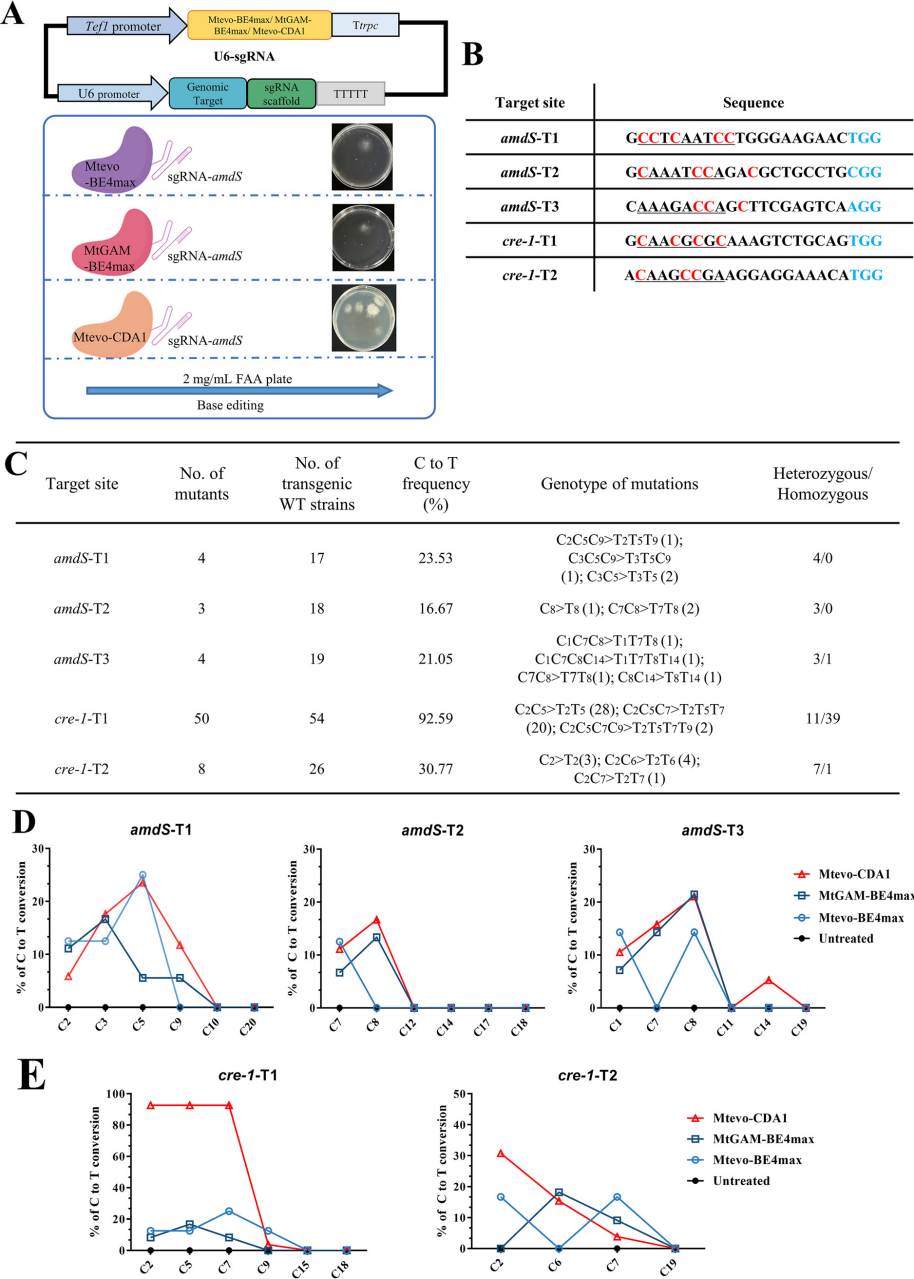
Comparison of C-to-T base editing by Mtevo-BE4max, MtGAM-BE4max, and Mtevo-CDA1. (A) The sgRNA expression cassette and schematic of base editing of the target gene *amdS* mediated by Mtevo-BE4max, MtGAM-BE4max, Mtevo-CDA1, and sgRNAs. (B) Target sites chosen for the genes *amdS* and *cre-1*. The PAM sites are colored blue. The potential editable window is underlined, and the predicted occurrence of edited Cs is colored red. (C) Frequencies of mutations induced by Mtevo-CDA1 in *amdS* and *cre-1* genes. (D, E) Frequencies of single C-to-T conversions by Mtevo-BE4max, MtGAM-BE4max, and Mtevo-CDA1 at three target sites in the *amdS* gene (D) and at two target sites in the *cre-1* gene (E).

**FIG 3:**
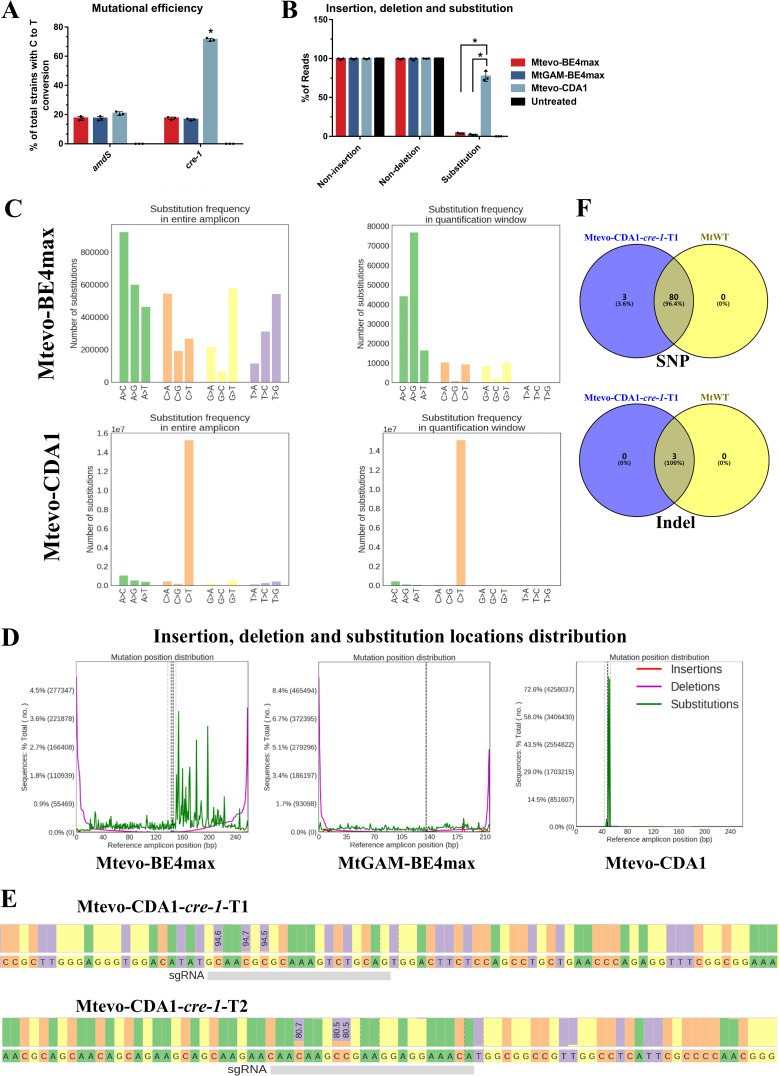
Comparison of editing accuracy and off-target effects among three different editors of the cytosine base-editing system. (A) Comparison of the mutation efficiency of *amdS* and *cre-1* mediated by three CBE editors (Mtevo-BE4max, MtGAM-BE4max, and Mtevo-CDA1). (B) Comparison of the proportions of insertions, deletions, and substitutions for three CBE editors (evo-BE4max, GAM-BE4max, and evo-CDA1) in 250-bp windows near target loci. (C) Mtevo-BE4max and Mtevo-CDA1 substitution frequencies in the entire amplicon and target window. The vertical coordinate shows the number of substitutions detected in the total sequencing data, and the horizontal coordinate shows the type of base substitution. The left shows the entire amplicon, and the right shows the target window. (D) The distribution of locations of insertions/deletions/substitutions for Mtevo-BE4max, MtGAM-BE4max, and Mtevo-CDA1 editors. Red represents insertion, purple represents deletion, green represents substitution, and the dotted line is the predicted editing site (sgRNA target). (E) Nucleotide percentage around sgRNA-*cre-1*-T1/T2. (F) Genome-wide off-target comparison of Mtevo-CDA1-*cre-1*-T1 and MtWT. The top shows SNP differences, and the bottom shows indel differences. Bars marked by asterisks in each group differ significantly from the unmarked bars (Tukey’s HSD; *, *P < *0.05). Error bars indicate the SD from multiple replicates.

The specific DNA single-strand breaks and C-T base substitutions produced by deaminase are mediated by the CBE system, resulting in loss of function of the target gene. To determine whether the CBE system was functional in *M. thermophila*, we designed sgRNA expression cassettes to target the *amdS* gene (44), which is essential for growth on acetamide as the only nitrogen source; the deletion resulted in resistance to fluoroacetamide (FAA). We combined Mtevo-BE4max, MtGAM-BE4max, and Mtevo-CDA1 with three U6p-*amdS*-sgRNAs (*amdS*-T1, *amdS*-T2, and *amdS*-T3, respectively) to form the expression cassettes. These cassettes were then delivered into protoplasts of the recipient *M. thermophila* strain M1 (8), which contained *amdS* and was sensitive to FAA (Fig. 2A). The CBE system can convert three different codons (CAA, CAG, and CGA) encoding two amino acids (glutamine [Gln] and arginine [Arg]) exclusively into the TAA, TAG, and TGA stop codons so that the target gene is inactivated by the introduction of a premature stop codon (22). The protospacer sequence targeted to *amdS* is shown in Fig. 2B. The mutations in the target region of *amdS* with FAA-resistant transformants were verified by DNA sequencing. As shown in Fig. 3A, the three editors had different efficiencies for the *amdS* gene, with Mtevo-BE4max at 17.4%, MtGAM-BE4max at 17.0%, and Mtevo-CDA1 showing the highest base-editing efficiency with an average of 20.4%. Moreover, we generated 11 Mtevo-CDA1-mediated C-T-base-mutant strains, including four strains at site sgRNA-*amdS*-T1, three strains at site sgRNA-*amdS*-T2, and four strains at site sgRNA-*amdS*-T3 (Fig. 2C). As shown in Fig. S2, the Mtevo-CDA1 produced C-T mutations in the FAA-resistant transformants at the sites of the *amdS*-T1, *amdS*-T2, and *amdS*-T3 through DNA sequencing of the target loci. These resulted in inactivation of the target gene *amdS*. Together, these results demonstrate that delivery of the CBE system and sgRNA cassettes can efficiently mediate mutation of the target gene via base substitutions in *M. thermophila*.

Given that the target *amdS* was an exogenous gene, to further evaluate the editing ability of the CBE system in *M. thermophila*, we selected the endogenous gene cre-1 as the target gene. The carbon catabolism repressor (CCR) *cre-1* (45) is a well-characterized transcription factor in filamentous fungi. We combined Mtevo-BE4max, MtGAM-BE4max, and Mtevo-CDA1 with two U6p-*cre-1*-sgRNAs (*cre-1*-T1 and *cre-1*-T2) to form expression cassettes. These cassettes were delivered into protoplasts of the recipient MtWT strain. The sequence of the protospacer region targeting *cre-1* is shown in Fig. 2B. On the basis of genomic PCR analysis and sequencing using specific primer sets, 4 of the 22 randomly selected Mtevo-BE4max-mediated transformants produced C-T substitutions at the protospacer-adjacent motif (PAM), all of which were heterozygous. The editing efficiency was only 18.2%. The editing effect based on the MtGAM-BE4max editor was even worse. Only 6 of the 35 randomly selected transformants produced base substitutions (17.1%) (Fig. 3A and Fig. S3). Interestingly, when using the Mtevo-CDA1 editor, 58 of the 80 randomly selected transformants had C-T base substitutions of the target, of which 40 were homozygous and 18 were heterozygous (Fig. 2C). The base-editing efficiency of Mtevo-CDA1 was the highest (72.5%), approximately 4-fold and 4.2-fold higher than those of Mtevo-BE4max (18.2%) and MtGAM-BE4max (17.1%), respectively (Fig. 3A and Fig. S3). Among them, Mtevo-CDA1 produced C-T mutations in 50 of 54 randomly selected transformants at the *cre-1*-T1 locus, with a high efficiency of up to 92.6%, although the editing efficiency of C-T at the cre-1-T2 locus was 30.77% (Fig. 2C). These results demonstrate that Mtevo-CDA1 has the highest editing efficiency among the three base editors in *M. thermophila*. When targeting the *amdS* gene and *cre-1* gene, we found that different target sites of the gene affected the editing efficiency, indicating that sgRNAs had large variations in efficiency. This difference can be partially attributed to the sgRNA sequence features and chromatin accessibility of the CRISPR system at the target site (46).

To evaluate the editing window size and the editing efficiency per point of CBE base editing in *M. thermophila*, we compared the above-mentioned three sites of the *amdS* gene and two sites of the *cre-1* gene. Transformants expressing only the Mtevo-CDA1 editor without sgRNA were used as a control. By analyzing the editing efficiency at every protospacer position across all 20 target sites, we found that the deamination window for Mtevo-CDA1 spanned 9 nt, from protospacer positions 1 to 9 (only one is mutated at position 14) compared with positions 1 to 17 for Mtevo-BE4max and MtGAM-BE4max (Fig. 2D and E). In addition, no C-T mutations were observed when using the Mtevo-CDA1 editor without sgRNA. The above results demonstrate the high editing efficiency and small editing window of the Mtevo-CDA1 editor.

### Editing accuracy and off-target effects between different editors of the cytosine base-editing system.

In view of the differences in functions of the three editors, the editing efficiency and accuracy of three editors (Mtevo-BE4max, MtGAM-BE4max, and Mtevo-CDA1) on the target sites of the *cre-1* gene were compared through amplicon deep sequencing. The C-to-T base editing of the *cre-1* gene was assessed by deep sequencing with 200,000 to 16,000,000 reads per locus (Fig. 3). Amplicon deep sequencing showed that all three editors hardly introduced insertions and deletions, and Mtevo-CDA1 had the highest base-editing substitution efficiency (72.6%), approximately 20-fold and 42-fold higher than those of Mtevo-BE4max (3.6%) and MtGam-BE4max (1.7%), respectively (Fig. 3B and Fig. S3). In the target editing window, the Mtevo-CDA1 editor only generated base substitutions inside the editing window and no editing outside, while there were many mutations outside the window that occurred in Mtevo-BE4max and MtGam-BE4max, indicating high editing specificity of the Mtevo-CDA1 editor (Fig. 3D). The editing accuracy of the Mtevo-CDA1 editor was also very high (Fig. 3C). In the region of 200 to 250 bp around the editing window, target C was replaced exclusively by T, and there were few other unexpected editing substitutions (Fig. 3D).

Whole-genome sequencing (WGS) results showed that MtWT and Mtevo-CDA1-*cre-1*-T1 produced 80 and 83 SNPs, respectively, compared with the reference genome (Fig. 3F). Both MtWT and Mtevo-CDA1-*cre-1*-T1 strains produced the three indels. Compared with MtWT, the Mtevo-CDA1-*cre-1*-T1 strain generated only three SNPs, and all mutations were C to T in the target editing site. The Mtevo-CDA1-*cre-1*-T1 strain produced no additional indels compared with the MtWT. These results suggested that Mtevo-CDA1*-cre-1*-T1 did not cause off-target effects across the genome (Fig. 3F).

We next evaluated the editing capability of different sgRNAs and Mtevo-CDA1 editor combinations. The deep sequencing results showed that the editing efficiency of C2, C5, and C7 of the protospacer sequence by using sgRNA-*cre-1-*T1 reached up to 94.6%, 94.7%, and 94.6%, respectively (Fig. 3E). The sequence upon using sgRNA-*cre-1-*T2 showed that the editing efficiency of C-T at C2, C6, and C7 reached 80.7%, 80.5%, and 80.5%, respectively (Fig. 3E). Although the use of different sgRNAs can cause differences in editing efficiency, for example, sgRNA-*cre-1-*T2 is not as highly efficient as sgRNA-*cre-1-*T1, a defect common to CRISPR systems, the combination of sgRNA-*cre-1*-T2 and the Mtevo-CDA1 editor can also successfully inactivate the *cre-1* gene. Efficient sgRNAs can also be predicted using machine learning.

Meanwhile, to verify whether different sgRNAs can cause unintended base pair mutations, insertions, and deletions, we conducted an in-depth analysis of the sequencing results to show the probability of base pair substitutions in the PAM region (evo-CDA1-*cre-1*-T1 and evo-CDA1-*cre-1*-T2 strains; Fig. S4). The obtained results demonstrated that C-T base substitutions efficiently occurred and that unexpected mutations were rarely found, such as C-G and C-A, regardless of whether sgRNA-*cre-1*-T1 or sgRNA-*cre-1*-T2 was used (Fig. S4A). Moreover, deep sequencing of the amplicons revealed no detectable insertions and deletions in the 200- to 250-bp region near the target loci with the corresponding sgRNA-*cre-1*-T1 or sgRNA-*cre-1*-T2 (Fig. S4B). Taken together, these findings indicated that our Mtevo-CDA1 editor is a promising tool for targeting single-base editing in manipulating the fungal genome.

### Inactivation of *cre-1* led to increased cellulase production.

**FIG 4:**
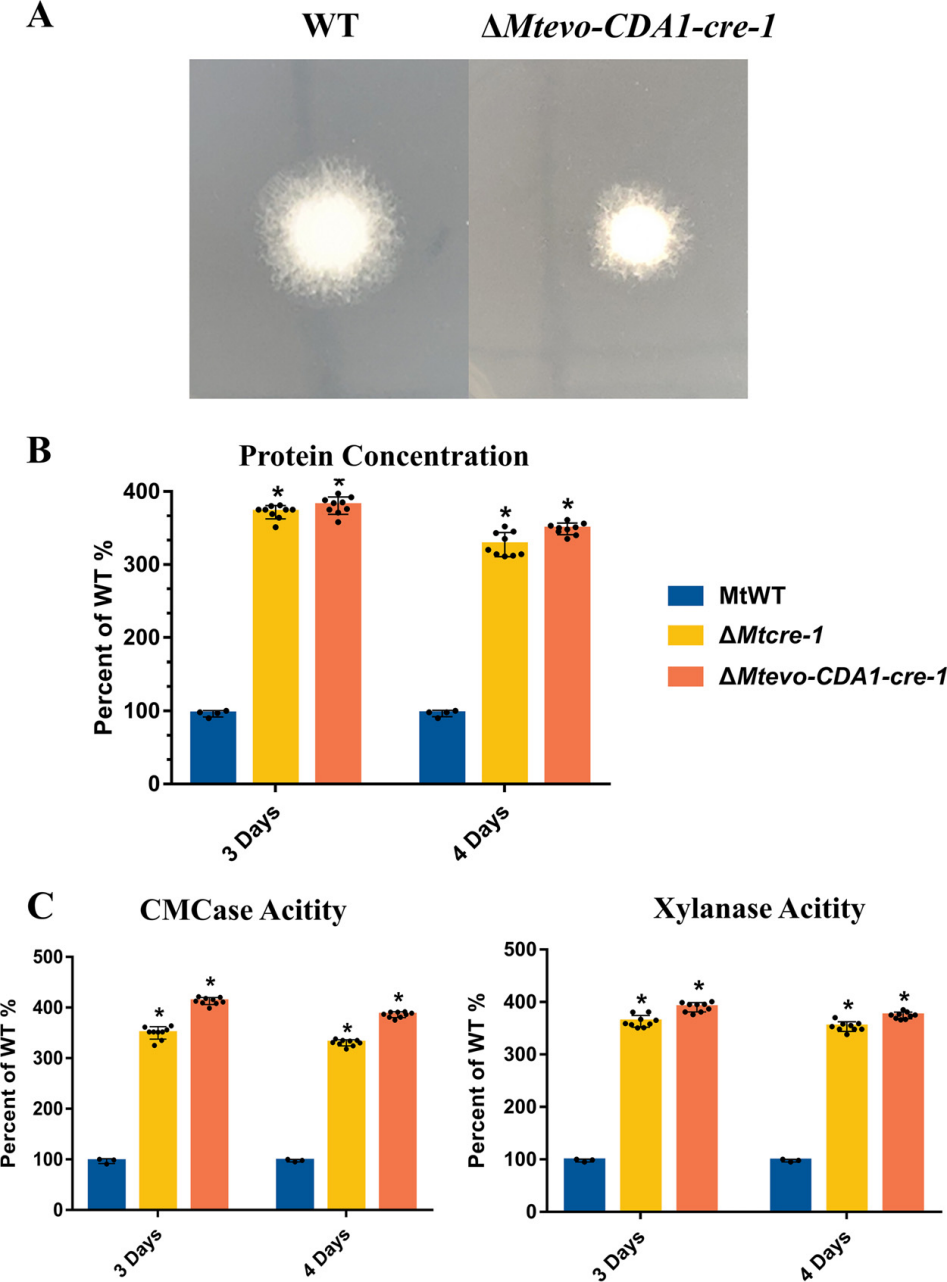
Phenotypic analysis of Δ*Mtcre-1*, Δ*evo-CDA1-cre-1*, and MtWT strains. (A) Δ*evo-CDA1-cre-1* and MtWT of *M. thermophila* on minimal medium plates after 2 days. (B, C) Assays for protein concentration and xylanase and CMCase activities of Δ*Mtcre-1*, Δ*evo-CDA1-cre-1*, and MtWT strains in 2% Avicel inducing medium after 4 days of culture. Each black dot represents an individual transformant. Bars marked by asterisks in each group differ significantly from the unmarked bars (Tukey’s HSD; *, *P < *0.05). Error bars indicate the SD from multiple replicates.

The cytosine base editor can efficiently cause C-T base substitutions; therefore, we chose the well-characterized gene *cre-1* to test whether gene function could be disrupted by the introduction of a stop codon using a base editor. *M. thermophila* CBE-mediated edited *cre-1* mutants were generated by our Mtevo-CDA1 editor and sgRNA-*cre-1-*T1 as described above (Fig. 3E). The *cre-1*-edited mutant strains (Δ*Mtevo-CDA1-cre-1*) exhibited slower-growing, denser hyphae on sucrose plates than MtWT (Fig. 4A), similar to the strain with deletion of the whole *cre-1* gene (Δ*Mtcre-1*) of *M. thermophila* generated by the CRISPR/Cas9 system in a previous study (8, 47–51). As expected, cellulase and hemicellulase production and activities in the *cre-1*-edited mutant strain (Δ*Mtevo-CDA1-cre-1*) were comparable to those in the mutant strain with *cre-1* deletion (Δ*Mtcre-1*; Fig. 4B and C). Therefore, the cytosine base editor can be efficiently used for gene inactivation by introducing a premature stop codon in thermophilic fungi.

### Investigation of the gene functions of *Mtclr-2* in *M. thermophila* using the base editor and classical CRISPR/Cas9 systems.

**FIG 5:**
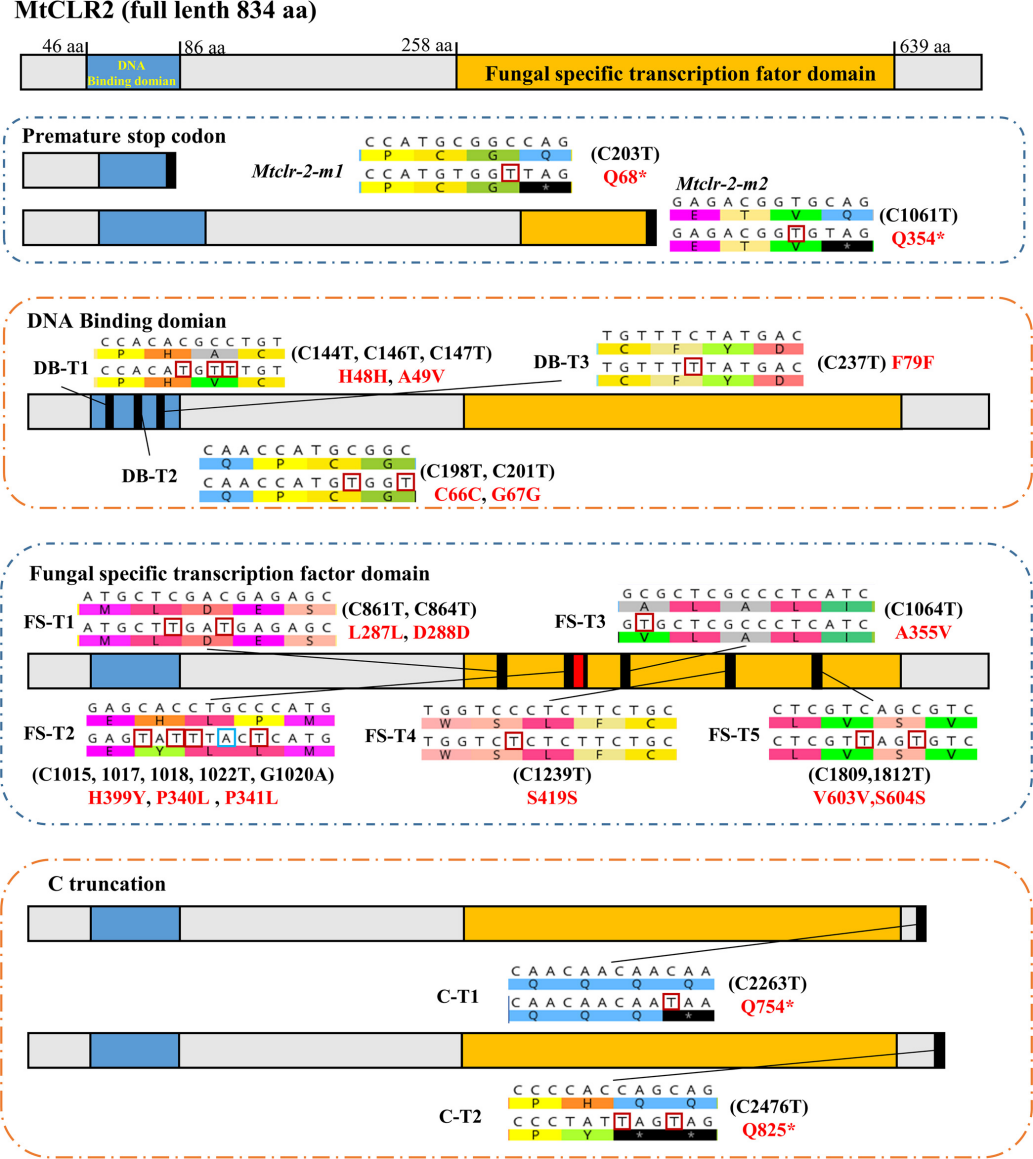
Schematic diagram of Mtevo-CDA1-mediated editing of the MtCLR-2 targeting region. The entire rectangular shape indicates the complete MtCLR-2 protein (full-length, 834 aa). The DNA-binding domain is in blue, and the fungus-specific transcription factor domain is in yellow. There are four different types of mutant strains: stop codon-edited mutants (*Mtclr-2*-m1 and *Mtclr-2*-m2), DNA-binding domain-edited mutants (DB-T1, DB-T2, and DB-T3), fungus-specific transcription factor domain-edited mutants (FS-T1, FS-T2, FS-T3, FS-T4, and FS-T5), and C-terminal-truncated mutants (C-T1 and C-T2).

**FIG 6:**
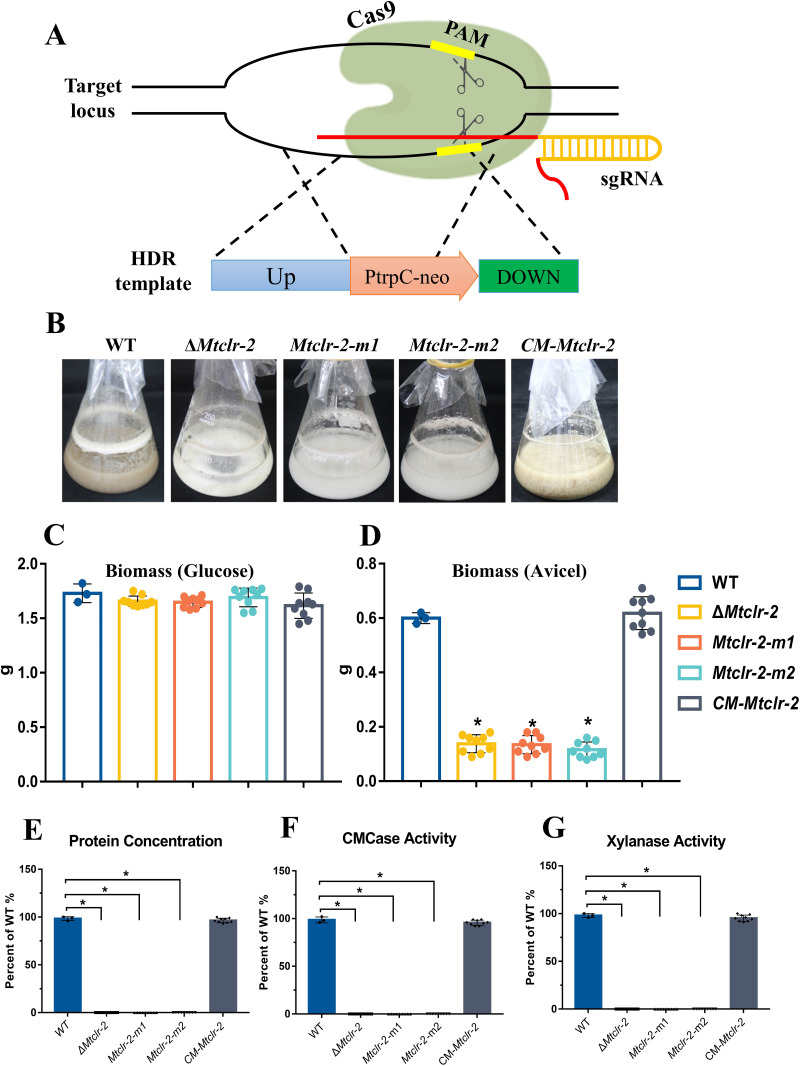
Protein production and enzyme activity phenotypes of *Mtclr-2* deletion mutants, complementation strains, and stop codon mutants. (A) Schematic of homologous recombination (HR) of the target gene *Mtclr-2* mediated by Cas9, gRNA, and donor DNA. (B) Phenotype of shake flask after 4 days of growth in 2% (wt/vol) Avicel medium. (C, D) Biomass of Δ*Mtclr-2*, complementation strains (*CM-Mtclr-2*), stop codon mutants (*Mtclr-2-m1* and *Mtclr-2-m2*), and MtWT from cultures on 2% glucose (C) after 4 days and 2% Avicel (D) after 4 days. (E) Assays of proteins secreted into supernatants of cultures of *M. thermophila* strains after 4 days of growth in 2% (wt/vol) Avicel medium. (F, G) Activities of CMCase and xylanase in culture supernatants of *M. thermophila* strains after 4 days of growth in 2% (wt/vol) Avicel medium. Each black dot represents an individual transformant. Bars marked by asterisks in each group differ significantly from the unmarked bars (Tukey’s HSD; *, *P < *0.05). Error bars indicate the SD from multiple replicates.

To further validate the application of the CBE in the genetic modification of thermophilic fungi, we chose the ortholog of essential cellulolytic regulators NcCLR-2 as a proof of concept, which has not yet been studied in *M. thermophila*. NcCLR-2 has been found to be a very important transcription factor for cellulase expression (52). We created its ortholog (XP_003660372, named *Mtclr-2*) mutant strains by introducing a premature stop codon at the N terminus (edited strain, Fig. 5). The mutations in the target region of *Mtclr-2* were verified by DNA sequencing (Fig. S5A). Two edited mutant strains were obtained in which C-T mutations occurred at nucleotide positions 203 and 1060 bp relative to the ATG of the *Mtclr-2* gene (Fig. S5A). Then, both mutations generated premature stop codons. These mutations generated truncated proteins with only 68 or 354 amino acids (aa) at the N terminus in each edited mutant, named *Mtclr-2-m1* and *Mtclr-2-m2*, respectively. We performed Western blotting analyses of intracellular protein from the MtWT and complementation strain *CM*-*MtCLR-2* and truncated *Mtclr-2* mutants, including *Mtclr-2*-*m1* and *Mtclr-2*-*m2*, to demonstrate the truncated MtCLR-2 protein. The rabbit polyclonal antiserum of MtCLR-2 with amino acids 92 to 275 at the N terminus was used in Western blotting analyses. As expected, the MtWT and *CM*-*MtCLR-2* strains showed a band at about 92 kDa with an expected size for intact MtCLR-2, while this protein band was not detected in the *Mtclr-2*-*m1* and *Mtclr-2-m2* mutants (Fig. S6B). The truncated protein in *Mtclr-2*-*m1* was only 68 amino acids at the N terminus with only 7 kDa and cannot be detected by the MtCLR-2 antiserum because MtCLR-2 antiserum included the amino acids 92 to 275 at the N terminus. The *Mtclr-2*-*m2* mutant showed a band at about 40 kDa with an expected size for truncated MtCLR-2 with 354 amino acids at the N terminus (Fig. S6B). At the same time, the gene-coding region of *Mtclr-2* (2505 bp) in the *M. thermophila* wild-type strain (MtWT) was deleted via homologous replacement with a *neo* cassette by our CRISPR/Cas9 technology (deletion strain, Δ*Mtclr-2*; Fig. 6A). The deletion mutant was rescued via ectopic integration of the cassette of the *bar* marker and its full-length sequence with flanking regions (*Mtclr-2* gene, 6,077 bp), named *CM-Mtclr-2*.

A previous study in N. crassa and A. nidulans demonstrated that the deletion of CLR-2/CLR-B led to a deficiency of growth on Avicel, and protein secretion and enzyme activities were abolished in the Δ*clr-2*/Δ*clrB* mutant (41). Similar to N. crassa Δ*clr-2* and A. nidulans Δ*clrB* mutants, both Δ*Mtclr-2* and *Mtclr-2-m1/2* grew very poorly in shake flasks when Avicel was the sole carbon source (Fig. 6D), whereas Δ*Mtclr-2* and *Mtclr-2-m1/m2* grew normally on glucose (Fig. 6A to C). Both Δ*Mtclr-2* and *Mtclr-2-m1/2* showed no detectable secreted proteins or cellulase and xylanase activities in contrast to the MtWT strains under cellulolytic conditions, in accordance with previous reports on N. crassa Δ*clr-2* and A. nidulans Δ*clrB* mutants (Fig. 6E to G and Fig. S6A). These results similarly demonstrate that edited mutant strains generated using the cytosine base editor (CBE) have the same gene inactivation ability as deleted mutant strains generated in a manner mediated by classical CRISPR/Cas9 technology.

### Investigation of the function of each motif of MtCLR-2 by using the cytosine base-editing system in *M. thermophila*.

Two clear motifs can be found in MtCLR-2 using standard bioinformatic tools: the DNA-binding region (46 to 86 amino acids [aa], 136 to 258 bp, referred to as the DB region) and the fungus-specific transcription factor domain (258 to 639 aa, 772 to 1917 bp, referred to as the FS region). The DNA-binding motif can recognize and bind DNA to specifically bind to and activate the regulons of genes. The fungus-specific domain is a conserved region, present in multiple zinc finger transcription factor families in fungi, including CLR-2. However, the functions of the two fungus-specific motifs of MtCLR-2 in *M. thermophila* are currently unknown. Therefore, targeting these two regions in the *Mtclr-2* gene using our CBE system, the Mtevo-CDA1 editor with the ability to produce C-T mutation, we began to investigate their detailed functions by designing three and five sgRNAs for the DB and FS regions, respectively (Table S2). Transformation of the Mtevo-CDA1 plasmid alone was used as a control. The mutations in the target region of Mtclr-2 were verified by DNA sequencing (Fig. S5).

**FIG 7:**
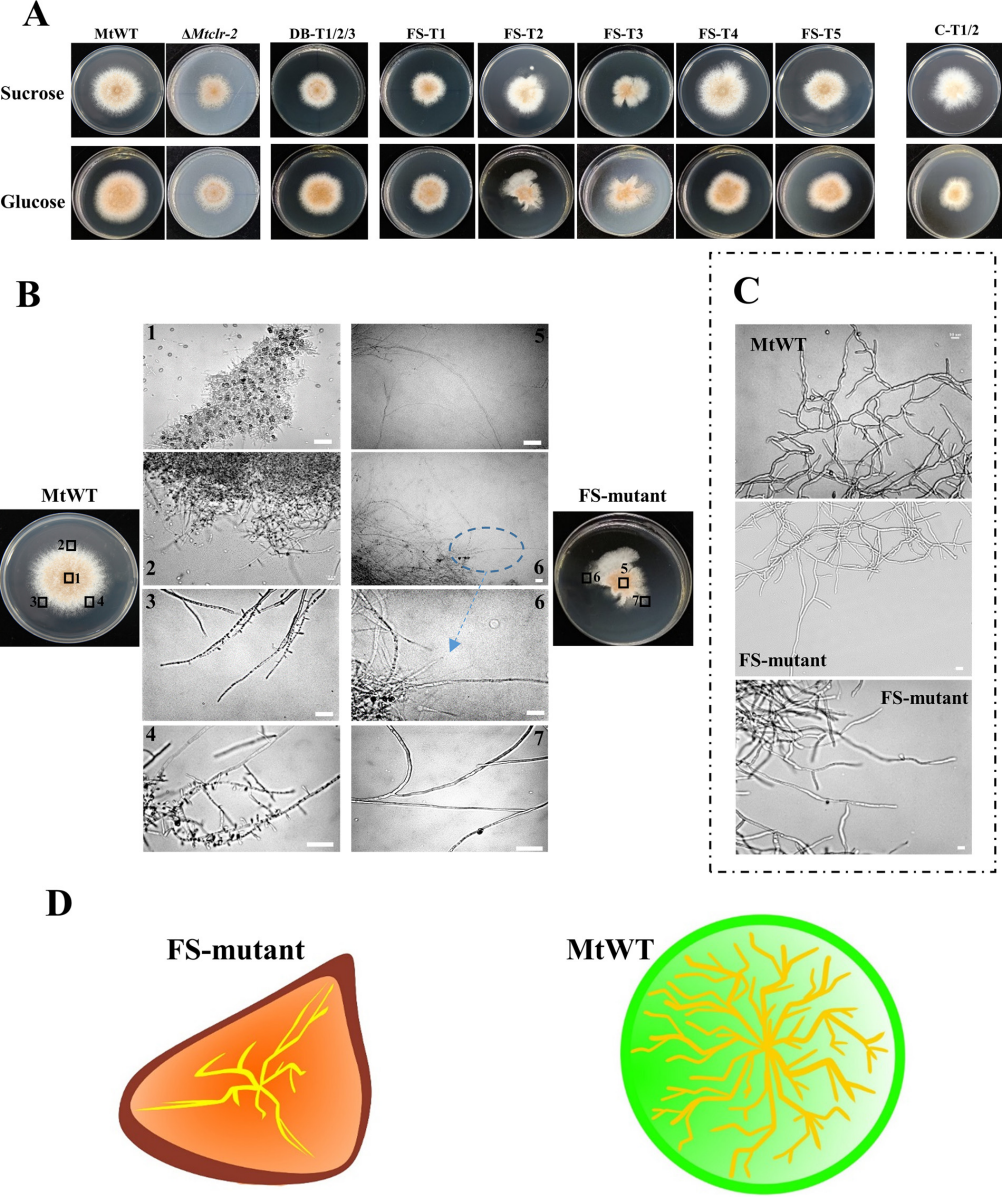
Growth comparison of MtCLR-2-mutant strains. (A) Colony growth and sporulation of Δ*Mtclr-2*, mutant strains (DB, FS, and C-terminal-truncated), and MtWT strains on minimal medium and sucrose medium plates after 3 days of culture. (B, C) Hyphal morphology of MtWT and FS mutants (FS-T2 and FS-T3). MtWT and FS mutants were grown in minimal solid plates with 2% (wt/vol) sucrose as the sole carbon source for 72 h (B), while MtWT and FS mutants were grown in liquid medium with 2% (wt/vol) sucrose as the sole carbon source for 24 h (C). Samples were observed under a laser scanning confocal microscope (Leica TCS SP5 II); scale bars, 10 μm. (D) Schematic diagram of mycelial development altering the ability to maintain colony morphology.

**FIG 8:**
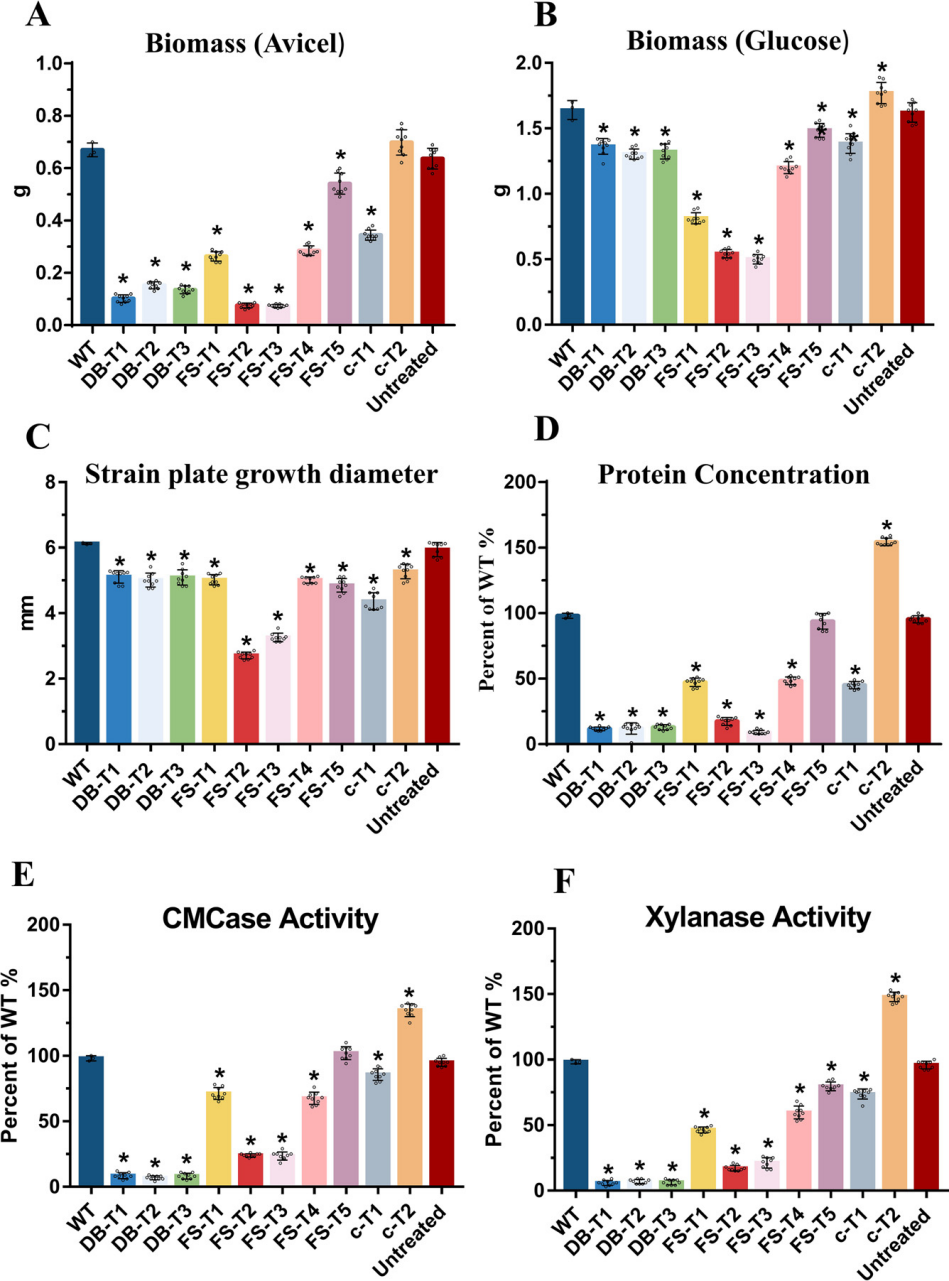
Phenotypic analysis of mutant strains of MtCLR-2. (A, B) Biomass of Δ*Mtclr-2*, mutant strains (DB, FS, and C-terminal-truncated), MtWT, and untreated strains (only evo-CDA1) from cultures on 2% Avicel (A) after 4 days and 2% glucose (B) after 2 days. (C) Strain plate growth diameter of Δ*Mtclr-2*, mutant strains (DB, FS, and C-terminal-truncated), MtWT, and untreated strains (only evo-CDA1) on minimal medium plates after 4 days of culture. (D–F) Assays for protein concentration (D) and CMCase (E) and xylanase (F) activities of Δ*Mtclr-2*, mutant strains (DB, FS, and C-terminal-truncated), MtWT, and untreated strains (only evo-CDA1) in 2% Avicel inducing medium after 4 days of culture. Each black dot represents an individual transformant. Bars marked by asterisks in each group differ significantly from the unmarked bars (Tukey’s HSD; *, *P < *0.05). Error bars indicate the SD from multiple replicates.

The results showed that mutant strains with C-to-T substitutions in the DB region were successfully obtained, namely, DB-T1, DB-T2, and DB-T3 (Fig. 5 and Fig. S5B). The DB-T1 mutant had C-to-T substitutions at positions 144, 146, and 147 (amino acid mutations H48H and A49V), and the DB-T2 mutant had C-to-T substitutions at positions 198 and 201 (amino acid mutations C66C and G67G). Base editing of the C-to-T conversion occurred at position 237 (amino acid mutation F79F) in the DB-T3 mutant. The two mutants DB-T2 and DB-T3 featured synonymous mutations. Interestingly, all the DB-mutated strains, including two strains with synonymous mutations, grew slower than the MtWT strain on glucose and sucrose medium plates, which was similar to the phenotype of the knockout strain Δ*Mtclr-2* (Fig. 7A). In addition, in terms of cellulase secretion capacity, DB-mutated strains had significantly reduced levels of protein secretion and azo-carboxymethyl cellulose (CMCase)- and xylanase activities, which were similar to those of deletion (Δ*Mtclr-2*) and edited mutants (*Mtclr-2-m1/2*). This indicated that the DB motif is essential for the function of MtCLR-2 (Fig. 8D to F). Because these mutants grew very poorly in shake flasks when Avicel was the sole carbon source, these phenotypes were as expected. Furthermore, regarding how the synonymous mutations of the mutant strains DB-T2 and DB-T3 affected gene function, we performed quantitative real-time PCR (qRT-PCR) assays using four pairs of the qPCR primers at different positions designed considering the gene truncation. The qRT-PCR results showed that the gene expression levels of the DB motif mutant strains were much lower than those of MtWT under the Avicel conditions, which proved that the sequence feature of the binding region was important for maintaining the normal functions of this transcription factor (Fig. S6C).

For the FS region, the Mtevo-CDA1 editor with the corresponding five sgRNAs targeting the FS region successfully created the C-T mutation strains FS-T1, FS-T2, FS-T3, FS-T4, and FS-T5 (Fig. 5 and Fig. S5C). These five FS-edited mutant strains can be divided into two types: one with synonymous mutations (FS-T1, FS-T4, and FS-T5) and the other with nonsynonymous mutations (FS-T2 and FS-T3; Fig. 5). The morphology of the mutant strains with synonymous mutations (FS-T1, FS-T4, and FS-T5) displayed no significant difference compared with the MtWT strain (Fig. 7A), but the protein yield and enzyme activities were lower than those of the MtWT strain (Fig. 8D to F). For nonsynonymous mutant strain FS-T2, multiple C-to-T or G-to-A mutations occurred within the editing window, which caused several amino acid changes in the motif (H399Y, P340L, and P341L). For the other nonsynonymous mutant strain FS-T3, the conversion of GCG to GTG occurred in the editing site, which caused the mutation of amino acid 355 from alanine (A) to valine (V) (Fig. 5). Surprisingly, FS-T2 and FS-T3 mutants grew much slower than MtWT, and their biomass growth in glucose and Avicel medium was reduced by ~65% (Fig. 8A to C). Notably, there were significant differences in the colony morphology of FS-T2 and FS-T3 strains on glucose and sucrose plates. As shown in Fig. 7A and Fig. S7, the hyphae of the FS-T2 and FS-T3 mutants lost their ability to maintain regular circular colonies and displayed irregular shapes. Microscopic observation revealed that the MtWT hyphae had many branches, and each part of the hyphae of the colony extended evenly. In the MtWT strain, spores were normally produced, and the colony shape was a regular circle. However, the hyphae of the FS-T2 and FS-T3 mutant strains had fewer branches, and some of them had no branches, in which just one trunk hypha extended very far and there were no surrounding secondary small hyphae, in contrast to the case in MtWT (Fig. 7D). The hyphae of colonies in different parts extended unevenly, and the ability to produce spores was significantly reduced at the same time. Together, these findings clearly showed that mutation in the fungus-specific motif, even one amino acid change, can cause significant developmental defects in conidiation, hyphal branching, and colony shape maintenance in thermophilic fungi. These hyphal phenotypes with fewer branches were also observed in the FS mutant strains grown in liquid culture (Fig. 7A to C).

In terms of the capacity to secrete cellulase, the amount of protein secreted by the strains with mutation in the FS motif decreased to various degrees (Fig. 8D to F). Among them, the strains with mutations in FS-T2 and FS-T3 amino acids had the most significant decreases in protein secretion capacity, which was only approximately 20% of that of MtWT when grown on Avicel medium for 4 days (Fig. 8D). The CMCase and xylanase activities were reduced by approximately 75% compared with those of the MtWT strain (Fig. 8E and F). These data indicated that FS mutant strains with different sequence mutation regions have defects in protein secretion of different degrees of severity, which were probably the effect of the very low growth on Avicel. In comparison with the DB mutant strains, the FS mutant strains still maintained a certain ability to degrade cellulose, which suggested that the DB motif is very essential in MtCLR-2.

### C-terminal truncation of the MtCLR-2 transcription factor led to increased cellulase production.

A previous report suggested that removing the C-terminal segment of the ACE3 regulator increased cellulase production in *T. reesei* (53). Comparative analysis of NcCLR-2 and MtCLR-2 revealed an extra segment in the C terminus of MtCLR-2 compared with its ortholog in N. crassa after 722 aa. To investigate whether this extra segment exerts an additional function, the C terminus of MtCLR-2 was truncated via the Mtevo-CDA1 base editor system, and two different stop codon mutant strains (C-T1 and C-T2) were obtained (Fig. 5). The mutant strain C-T1 had one C-to-T conversion at position 2637, resulting in mutation of amino acid 754Q into a stop codon, while the C-T2 mutant had a C-to-T substitution at position 2476, resulting in mutation of amino acid 825Q into a stop codon. Eighty-two and 12 C-terminal amino acids were removed in mutant strains C-T1 and C-T2, respectively (Fig. 5 and Fig. S5D). There was no significant difference in the growth of the mutant C-T1 compared with the MtWT strain (Fig. 7A), while the biomass of the C-T2 strain increased by approximately 20% compared with that of MtWT in both glucose and Avicel media (Fig. 8A to C). Cellulase production was affected by C-terminal truncation. Compared with MtWT, cellulase secretion in the C-T1 mutant was reduced to 54%, whereas cellulase production in the C-T2 mutant was surprisingly increased by 40% (Fig. 8D to F). The expression levels of the MtCLR-2 target genes, including the major cellulases *Mtcbh-1* (MYCTH_109566), *Mtcbh-2* (MYCTH_66729), *Mtgh5-1* (MYCTH_86753), and *Mtgh61-7* (AA9, MYCTH_110651), were measured in the C-T2 mutant and MtWT by qRT-PCR under cellulolytic conditions. These four genes showed increased expression levels (approximately 1.5~2.2-fold) in the C-T2 mutant compared to MtWT when exposed to Avicel (Fig. S6D). These data indicated that the increase in the expression levels of the major cellulase genes in the 12-amino-acid truncation of the MtCLR-2 C-terminal mutant might the partial cause of the enhanced cellulase production phenotype. These results indicated that the C terminus of MtCLR-2 is important for its functions in regulating lignocellulose-active gene expression. Interestingly, removing the 12 amino acids of the C terminus can significantly improve lignocellulase production, although additional experiments are needed to clarify the mechanism behind this.

## DISCUSSION

The CRISPR/Cas9 system has been established in many filamentous fungi, including N. crassa (54), A. oryzae (55), A. niger (56, 57), A. nidulans (56), A. fumigatus (58), *T. reesei* (59), and *M. thermophila* (44). The CRISPR/Cas9 genome-editing technology has been widely used for genetic engineering, such as gene insertion, deletion of DNA fragments, and multiple site mutations, but very few studies on single-base editing of DSBs in filamentous fungi have been performed. At present, only the established BE3 single-base-editing system has been successfully used in A. niger to edit the uridine synthesis gene *pyrG*, the pigment gene *fwnA*, and the nonphenotypic gene *prtT* (60). In this study, we constructed three new cytosine base editors for the first time in the thermophilic fungus *M. thermophila* and successfully edited three target genes *amdS*, *cre-1*, and *Mtclr-2.*

Compared with Mtevo-BE4max and MtGAM-BE4max, Mtevo-CDA1 has the highest base-editing efficiency (up to 92.6%), which is higher than that of animals (14) and plants (29) but lower than the editing efficiency of prokaryotic microorganisms (61, 62). However, the low base-editing efficiency of some target genes suggests that the selection of target genes has a large impact on the editing results. Meanwhile, the selection of sgRNA also limits the application of base editors, and to obtain efficient and applicable sgRNAs, we usually choose the first G in N20 combined with a machine learning approach (63). Furthermore, whole-genome sequencing showed that Mtevo-CDA1 is a fungal base editor with a low off-target rate (Fig. 3F). In conclusion, Mtevo-CDA1 is a better and more suitable single-base-editing tool for *M. thermophila* cytosines.

In cellulolytic fungi, lignocellulolytic enzyme production is regulated at both transcriptional (41) and posttranslational levels (64). For instance, misexpression of the transcription factors *cre-1* and *clr-2* can significantly influence cellulase production. The *cre-1* or *Mtclr-2* strains based on our previous CRISPR/Cas9 knockout system and the edited mutant strains generated by this newly established CBE inactivation system have similar colony morphology and protein secretion ability, indicating that the inactivation of genes based on the strategy of introducing a premature stop codon can achieve the same effect as knockout manipulation, and, because it did not produce DSBs, it is much less harmful to fungal strains. Our research also clearly demonstrated that MtCLR-2 has a conserved role in cellulase gene regulation in thermophilic cellulolytic fungi because it was also found in mesophilic fungi like N. crassa.

Targeting the MtCLR-2 protein, we explored the functions of regions within this protein, including the DNA-binding domain (DB region) and the fungus-specific domain (FS region), by taking advantage of the base editor that we developed here and generated C-to-T mutation in the targeted loci. All the DB-mutated strains grew slower than the MtWT strain on glucose and sucrose media plates, which was similar to the phenotype of the knockout strain Δ*Mtclr-2* (Fig. 7A). In addition, in terms of cellulase secretion capacity, DB-mutated strains had significantly reduced protein secretion, and CMCase and xylanase activities. Interestingly, even the synonymous mutations in the binding region caused the loss of normal functions, all of which were similar to the deletion strain Δ*Mtclr-2* (Fig. 6 to 8). The qRT-PCR results showed that the gene expression levels of the DB motif mutant strains were lower than those of MtWT, which indicated that the sequence integrity of the binding region might be important for maintaining normal gene functions (Fig. S5B in the supplemental material). Moreover, a previous study in N. crassa demonstrated that codon usage bias strongly correlates with protein and mRNA levels and that codon usage is an important determinant of gene expression (65). Therefore, the synonymous mutations of the mutants DB-T2 and DB-T3 equally affected the function of MtCLR-2, suggesting an important role of codon usage bias in the *M. thermophila* genome.

The functions of the fungus-specific domain (FS region) of MtCLR-2 were investigated systematically using our base editor, and the key motif region was identified at approximately 900 to 1100 bp, which encoded the region of 320 to 400 aa. The fungus-specific region in MtCLR-2 was shown to be critical for its function not only in cellulase gene expression but also in fungal development, including conidiation, hyphal branching, and colony shape formation, as found in the present study (Fig. 7). Hyphae are one of the most polarized cell forms (66–68). The ability of filamentous fungi to generate polarized cells with a variety of shapes reflects precise temporal and spatial control of polar axis formation. Loss of the ability to maintain circular colonies upon mutation of the FS region of CLR-2 indicated that the communication between the hyphae within the colony was damaged, which might have been caused by branching defects in these mutants. However, this phenomenon was barely explored; thus, further research is required to deepen our understanding.

Eighty-two and 12 amino acids were removed from the C terminus in the mutant strains C-T1 and C-T2, respectively, via the Mtevo-CDA1 base editor system. Cellulase production was significantly affected by these C-terminal truncations. Compared with the MtWT, cellulase secretion in the C-T1 mutant decreased to 54%, whereas that in the C-T2 mutant surprisingly increased by 40% (Fig. 8D to F). Together, these results indicated that the C terminus of MtCLR-2 is important for its functions in regulating lignocellulase gene expression. Interestingly, removing the 12 amino acids of the C terminus can significantly improve lignocellulase production, although further experiments are needed to illustrate the mechanism behind this.

### Conclusion.

Here, we successfully constructed a cytosine base editor in *M. thermophila* with high efficiency (up to 92.6%). Taking advantage of the CBE that we developed, the functions of the major cellulase transcription factor MtCLR-2 were systematically investigated, including the function of each motif of the protein. The fungus-specific motif of MtCLR-2 was found to be deeply involved in conidiation, hyphal branching, and maintenance of a circular colony shape.

## MATERIALS AND METHODS

### Strains and growth conditions.

*Myceliophthora thermophila* (ATCC 42464) was obtained from the American Type Culture Collection (ATCC). *M. thermophila* strains were cultured on Vogel’s minimal medium (MM) supplemented with 2% sucrose at 45°C for 7 days to obtain conidia. Antibiotics were added when needed to screen for transformants. For flask culture, *M. thermophila* conidia at 10^6^ mL^−1^ were inoculated in 100 mL of medium (containing 1× Vogel’s salt, 2% Avicel, and 0.75% yeast extract) at 45°C with shaking at 150 rpm. For vector manipulation and propagation, Escherichia coli DH5α (Invitrogen, Shanghai, China) was cultured at 37°C in Luria-Bertani broth with kanamycin or ampicillin (100 μg mL^−1^) for plasmid selection.

### Plasmid construction for genetic engineering.

All the primer sequences used in this study are listed in Table S1 in the supplemental material. All the PCR products were amplified using Phusion high-fidelity DNA polymerase (Thermo Fisher, Waltham, MA, USA). A codon-optimized nCas9-3.7 gene, the rAPOBEC1 gene, the PmCDA1 gene, and the gene encoding the uracil glycosylase inhibitor (UGI) protein were synthesized by Life Sciences Research Services (Genewiz, Suzhou, China). UGI, P*tef1* promoter (8), and T*trpC* terminators were amplified and assembled into the p0380-bar plasmid (69) using the NEB Gibson assembly kit to form evo-BE4max (Ptef1-NLS-rAPOBEC1-nCas9-3.7-2xUGI-NLS-TtrpC), GAM-BE4max (Ptef1-NLS-GAM-rAPOBEC1-nCas9-3.7-2xUGI-NLS-TtrpC), and evo-CDA1 (Ptef1-NLS-CDA1-nCas9-3.7-UGI-NLS-TtrpC) editors. The Cas9 expression PCR cassette bar-Ptef1-Cas9-TtprC was constructed previously, as were the selectable markers *neo* (GenBank HQ416708, *neo* gene for neomycin resistance protein) and *bar* (GenBank X17220, *bar* gene for phosphinothricin acetyltransferase). The sequences of each partial expression cassette are provided in Note S1.

To select for specific sgRNAs targeting *amdS* (GenBank M16371.1), *cre-1* (MYCTH_2310085), and *Mtclr-2* (MYCTH_38704), all sgRNA target sites in the genome of *M. thermophila* were identified using the sgRNACas9 tool (70), and sgRNA target sites with high scores were chosen (Table S2). A target-directed *M. thermophila* U6 promoter-driven sgRNA was created by overlapping PCR with the primers given in Table S1 and cloned into a pJET1.2/blunt cloning vector, which yielded the corresponding plasmids U6p-*amdS*-sgRNA, U6p-*cre-1*-sgRNA, and U6p-*Mtclr-2*-sgRNA (Note S2).

For the construction of gene deletion substrates, the PtrpC-neo cassette was amplified from a p0380-neo plasmid (44). The 5′- and 3′-flanking fragments of *Mtclr-2* were separately amplified from *M. thermophila* genomic DNA via PCR with paired primers (Table S2). The amplified 5′, 3′, and PtrpC-neo fragments were assembled and ligated into a pJET1.2/blunt cloning vector using an NEB Gibson assembly kit to generate the donor-*clr-2* DNA sequence.

### Expression of CBEs in *Myceliophthora thermophila*.

The PEG-mediated transformation of *M. thermophila* protoplasts was performed as described previously (45). For three CBE expression vectors (Mtevo-BE4max, MtGAM-BE4max, and Mtevo-CDA1), MtWT was used as the host strain (44). Briefly, 10 μg of the plasmid of the CBE expression vector (Mtevo-BE4max, MtGAM-BE4max, and Mtevo-CDA1) was transformed separately into MtWT protoplasts. Colonies grown for 4 days on MM at 35°C were screened for bar gene resistance using 100 μg mL^−1^ phosphinothricin (Sigma-Aldrich, St. Louis, MO, USA), followed by sequential identification via PCR analysis. The positive transformants from each construct were named OE-CBE (OE-Mtevo-BE4max, OE-MtGAM-BE4max, and OE-Mtevo-CDA1). OE-CBE-positive transformants selected as replicates were subjected to three consecutive rounds of subculture; their phenotypes, including secreted protein production, mycelial dry weight (biomass), and lignocellulosic enzyme activities, were examined in parallel with those of the MtWT strain.

### Transformation of *Myceliophthora thermophila* protoplasts.

Transformation of *M. thermophila* protoplasts was performed in accordance with a previously described procedure (45). The *amdS*-expressing strain M1 was constructed in the laboratory (8). The *amdS* mutant M1 was used as the host by transforming CBE editors (evo-BE4max, GAM-BE4max, and evo-CDA1) with guide RNA (gRNA)-*amdS*-T1/T2/T3 at the same molar concentration (10 μg) into M1 protoplasts. Possible transformants were selected on MM spiked with 100 μg mL^−1^ phosphinothricin, followed by sequencing identification by paired primers for each target site (Table S1).

For the *cre-1* and *Mtclr-2* genes, 10 μg of the CBE expression cassette with sgRNA-*cre-1* or sgRNA-*Mtclr-2* was added to the fungal protoplasts. Control experiments were performed by adding 10 μg of only the evo-CDA1 expression cassette without sgRNA to the fungal protoplasts. Transformants were screened for bar resistance with phosphinothricin (100 μg mL^−1^) or for neomycin resistance with G418 (80 μg mL^−1^; Beijing Solarbio Science & Technology Co., Ltd., Beijing, China), followed by sequencing identification with paired primers for each target site (Table S1).

For *Mtclr-2* gene knockout, 10 μg of the Cas9 expression PCR cassette bar-Ptef1-Cas9-TtprC, gRNA expression PCR cassette (U6p-*Mtclr2*-sgRNA), and donor-*Mtclr-2* were mixed at a molar concentration ratio of 1:1:1 and added to the fungal protoplasts. Transformants were screened for bar resistance with phosphinothricin (100 μg mL^−1^) and for neomycin resistance with G418 (80 μg mL^−1^), followed by PCR identification with paired primers (Table S1).

### Protein and enzyme activity measurements.

Protein concentration in the supernatants was determined using a Bio-Rad protein assay kit (Bio-Rad, Hercules, CA, USA). Absorbance was measured at 595 nm, and bovine serum albumin was used as the standard. For protein gel electrophoresis, 20 μL of unconcentrated culture supernatant was loaded onto a polyacrylamide gel (Novex NuPAGE Precast Protein Gels, Thermo Fisher Scientific) for sodium dodecyl sulfate-polyacrylamide gel electrophoresis (SDS-PAGE). Endoglucanase and endo-1,4-β-xylanase activity in the culture supernatants was determined using an Azo-CM-cellulose assay kit (Megazyme) and an Azoxylan kit (Megazyme), in accordance with the manufacturer’s instructions. All estimates were performed in triplicate assays. The statistical significance of differences between two conditions was analyzed using two-tailed Student’s *t* test. For all tests, significance was set at a *P *value of <0.01 (*).

### Amplicon deep sequencing and data analysis.

For amplicon deep sequencing and data analysis, genomic DNA was extracted from the fungal hyphae after 24 h in glucose medium and used as a template. In the first round of PCR, the target region was amplified using site-specific primers (Table S1). In the second round, both forward and reverse barcodes were added to the ends of the PCR products for library construction (Genewiz, China). Equal amounts of the PCR products were pooled, and samples were sequenced commercially (Genewiz, China) using the Illumina NovaSeq 6000 platform. The sgRNA target sites in the sequenced reads were examined for C-to-T substitutions and indels. Amplicon sequencing was repeated three times for each target site using genomic DNA extracted from three independent strains.

### Quantitative real-time qPCR.

The *M. thermophila* strains were inoculated into 1× Vogel’s salts with 2% (wt/vol) sucrose and grown for 16 h at 35°C. The mycelia of each strain were collected and washed three times with 1× Vogel’s salts and transferred into medium with 2% (wt/vol) Avicel as the carbon source for an additional 4 h of culture. Mycelia were harvested by vacuum filtration and immediately homogenized in liquid nitrogen before extracting total RNA with TRIzol reagent (Invitrogen). Then, qPCR was performed using the iScript cDNA synthesis kit and SYBR green real-time PCR master mix (Toyobo, Osaka, Japan), in accordance with the manufacturers’ instructions, on a CFX96 real-time PCR detection system (Bio-Rad, Hercules, CA, USA). Each reaction was performed in triplicate. The actin gene (MYCTH_2314852 for *M. thermophila*) was used as an endogenous control for all experiments. All primers used in this study are listed in Table S1. The transcript level of each gene was estimated using the cycle threshold (2^–ΔΔ^*^CT^*) method (71). The proportion of each gene transcript in each mutant relative to that in the MtWT strain was calculated as the relative transcript level.

### Generation of MtCLR-2 antiserum and Western blotting.

The method used to generate MtCLR-2 antiserum was similar to that described by Lin et al. (72). Briefly, the pGEX-4T-1 vector and Escherichia coli BL21(DE3) cells were used to express the glutathione *S*-transferase (GST)-MtCLR-2 (amino acids 92 to 275 at the N terminus) fusion protein. The purified recombinant protein was used as the antigen for immunizing rabbits, which generated rabbit polyclonal antisera, as previously reported (72). The strains cultured after a 4-h induction on 2% Avicel medium were collected for Western blotting analyses. The rabbit polyclonal antiserum and the goat anti-rabbit IgG horseradish peroxidase (HRP) antibody at a dilution of 1:1,000 were used as the primary and secondary antibodies (Abmart, Shanghai, China), respectively.

### Data availability.

All sequencing data supporting the findings of this study are available in the article. The genome sequencing and deep sequencing data have been deposited at the NCBI BioProject database under accession code number PRJNA826319.
