# Supplementary material for: Development of an Efficient C-to-T Base-Editing System and Its Application to Cellulase Transcription Factor Precise Engineering in Thermophilic Fungus Myceliophthora thermophila
Source: Microbiol Spectr. 2022 May 24;10(3):e02321-21. doi: 10.1128/spectrum.02321-21 (PMC9241923; doi:10.1128/spectrum.02321-21)
Supplement: SUPPLEMENTAL FILE 1 — Supplemental material. Download spectrum.02321-21-s001.pdf, PDF file, 1.7 MB [file spectrum.02321-21-s001.pdf]

Development of an efficient C-to-T base editing system and its application on cellulase transcription factors precise engineering in thermophilic fungus *Myceliophthora thermophila*

Chenyang Zhang[a,b,c], Nan Li[a,b,d], Lang Rao[a,b], Jingen Li[a,b], Qian Liu[a,b,]*, Chaoguang Tian[a,b,]*

[a]*Key Laboratory of Systems Microbial Biotechnology, Tianjin Institute of Industrial Biotechnology, Chinese Academy of Sciences, Tianjin 300308, China*

[b]*National Technology Innovation Center of Synthetic Biology, Tianjin 300308, China*

[c]*University of Chinese Academy of Sciences, Beijing 100049, China*

[d]*College of Biotechnology, Tianjin University of Science & Technology, Tianjin 300457, China.*

*Correspondence emails: Qian Liu, liu_q1@tib.cas.cn, and Chaoguang Tian, tian_cg@tib.cas.cn

List of Supporting Information

Fig. S1 Growth comparison of *M. thermophila* WT, OE-Mtevo-BE4max, OE-MtGAM-BE4max, and OE-Mtevo-CDA1. Plate growth diameter (A) and growth assay (B) on various carbon sources (1% w/v). All carbon sources were added at 1% for plate culture assays. Cultures were incubated at 45 ℃ for 4 days. All assays were performed with multiple culture replicates. Each black dot represents individual transformant.

Fig. S2 Sequencing peak map of the evo-CDA1 editor for the gene *amdS*.

Fig. S3 Sequencing peak map of the three CBE editors for the gene *cre-1*.

Fig. S4 Comparison of off-target effects between two sgRNAs of Mtevo-CDA1. (A) Two sgRNAs (sgRNA-*cre-1*-T1 and sgRNA-*cre-1*-T2) and Mtevo-CDA1 substitution frequency in the entire amplicon and quantification window. The vertical coordinate shows the number of substitutions detected in the total sequencing data and the horizontal coordinate shows the type of base substitution. The left panel shows the entire amplicon and the right panel shows the target window. (B) The distribution of locations of insertions, deletions, and substitutions for two sgRNAs (sgRNA-*cre-1*-T1 and sgRNA-cre-1-T2) of Mtevo-CDA1.

Fig. S5 Sequencing peak map of *Mtclr-2* gene mutant strains.

Fig. S6 Assays of the secreted protein concentration, western blotting and gene expression level in the MtCLR-2 mutants. (A) SDS-PAGE analysis of proteins secreted by Δ*Mtclr-2*, *Mtclr-2-m1*, *Mtclr-2-m1*, CM-*Mtclr-2* and MtWT strains after 4 days on 2% Avicel medium. (B) Western blotting of intracellular protein from *Mtclr-2-m1* and *Mtclr-2-m2* and MtWT strains after a 4-h induction on 2% Avicel medium and probed

with rabbit polyclonal antiserum. (C) Gene expression of *Mtclr-2* gene from the selected strains after a 4-h induction on 2% Avicel medium by using four pairs of the qPCR primers at different positions redesigned considering the gene truncation. (D) Gene expression levels of the major four MtCLR-2 target genes from the selected strains after a 4-h induction on 2% Avicel medium. Each black dot represents individual transformant. Bars marked by asterisks in each group differ significantly from the unmarked bars (Tukey's HSD, $*P < 0.05$). Error bars indicate the SD from multiple replicates.

Fig. S7 Colony growth and sporulation of mutant strains (FS-T2 and FS-T3) on minimal medium plates after 4 days of culture.

Table S1 Sequences of all the primers used in this study.

Table S2 All gRNAs used in this study.

Note S1 Sequences of CBE constructs.

Note S2 Sequences of U6-sgRNA.

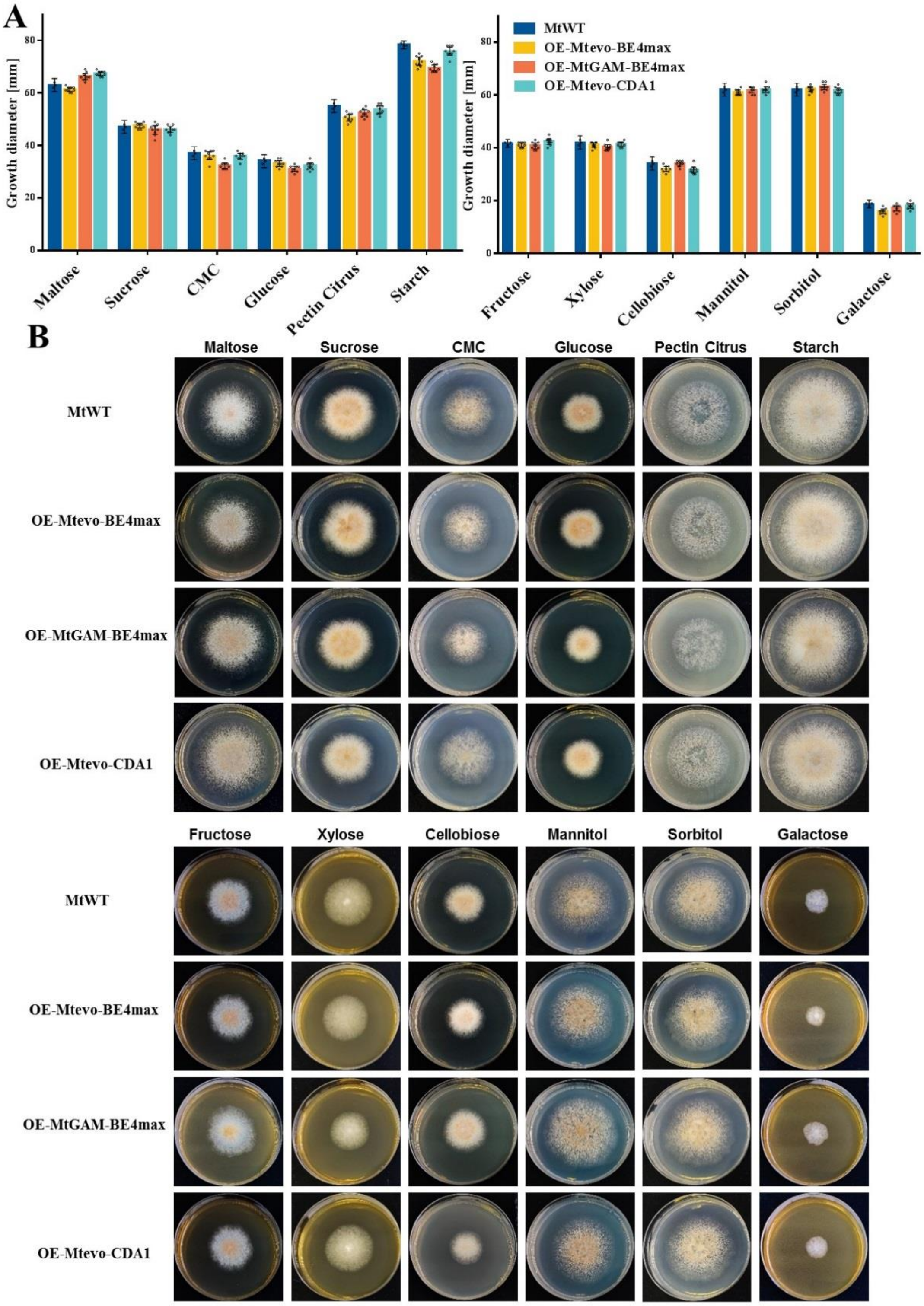

Fig. S1 Growth comparison of *M. thermophila* WT, OE-Mtevo-BE4max, OE-MtGAM-BE4max and OE-Mtevo-CDA1. Plate growth diameter (A) and growth assay (B) on various carbon sources (1% w/v). All carbon sources were added at 1 % for plate culture assays. Cultures were incubated

at 45 ℃ for 4 days. All assays were performed with multiple culture replicates. Each black dot represents individual transformant.

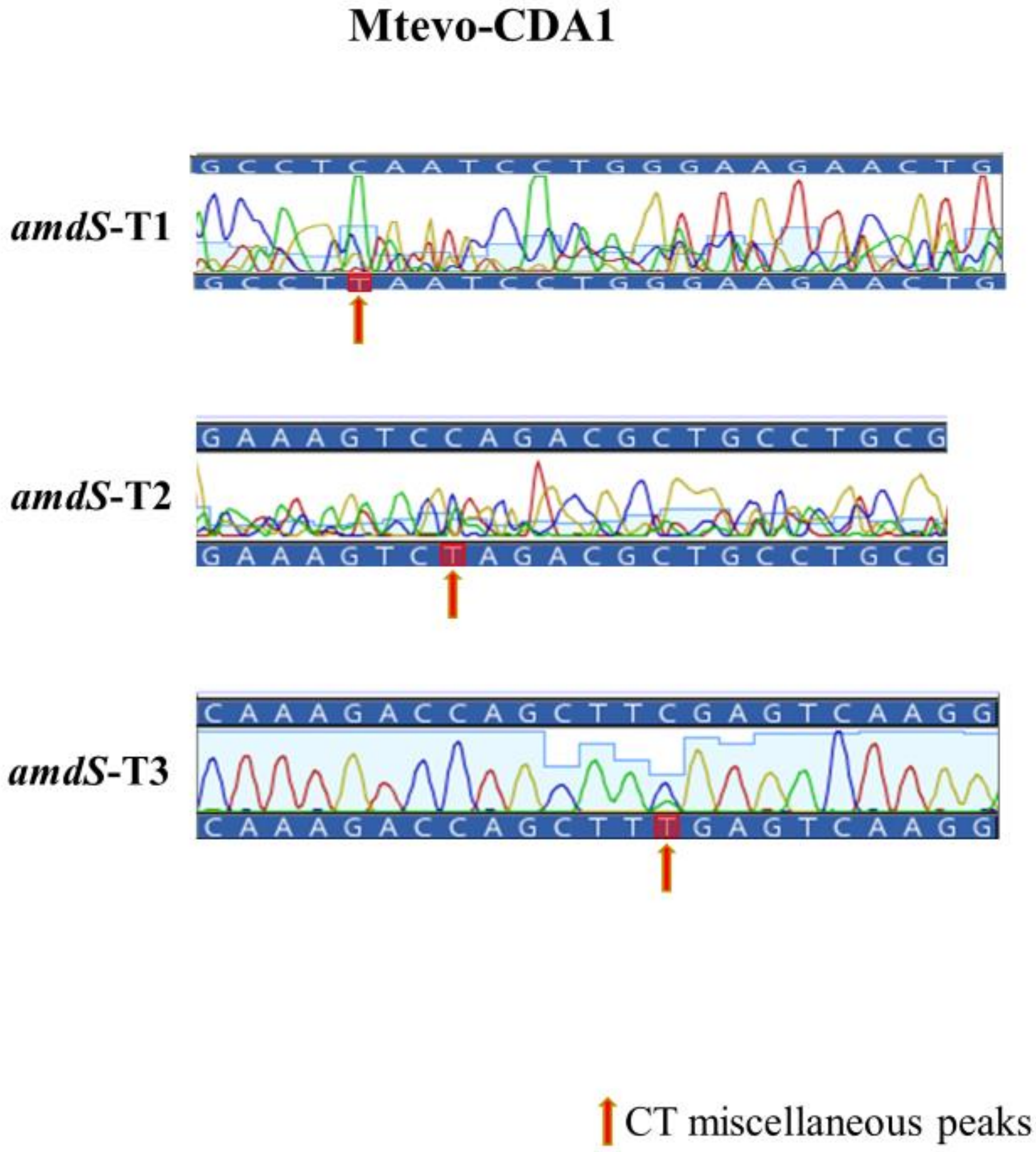

Fig. S2 Sequencing peak map of the evo-CDA1 editor for the gene *amdS*.

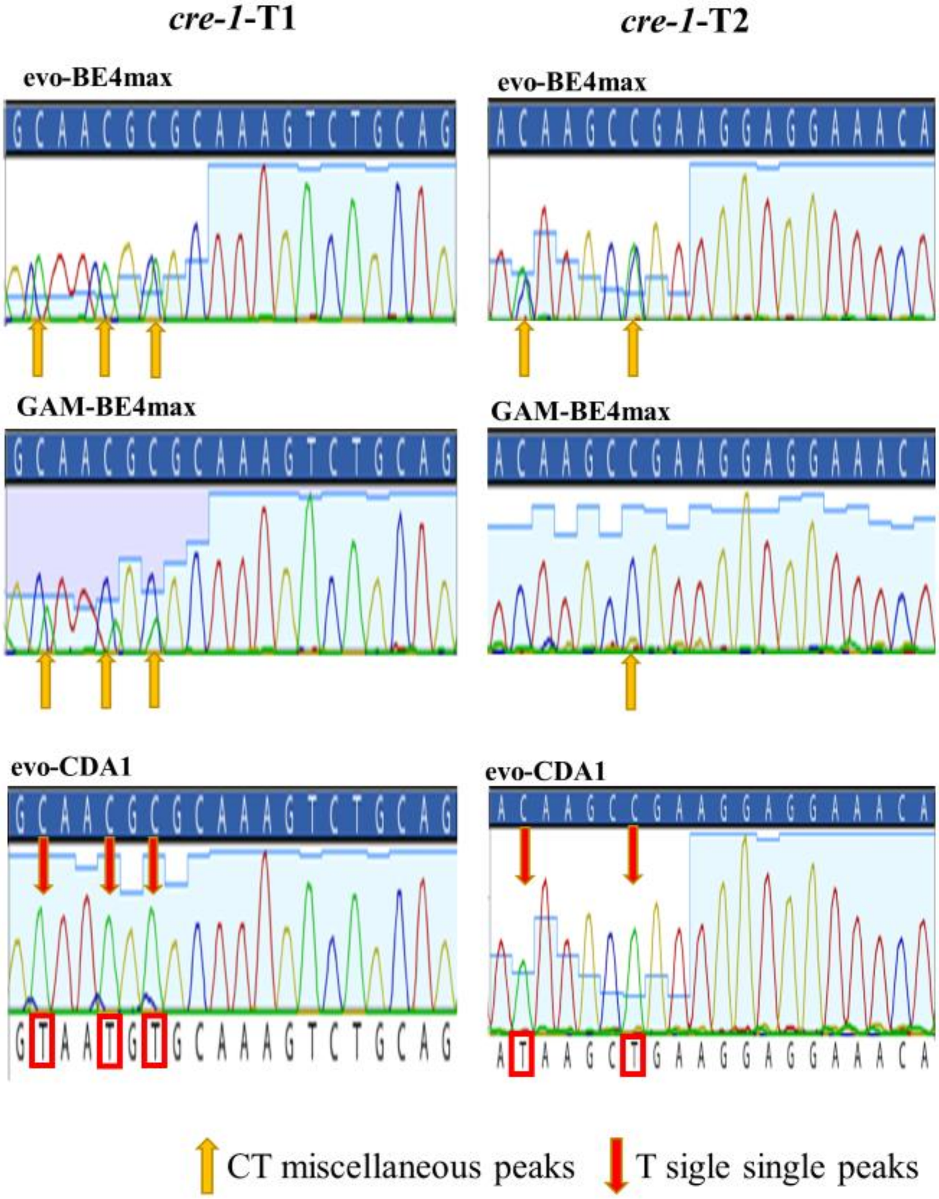

Fig. S3 Sequencing peak map of the three CBE editors for the gene *cre-1*.

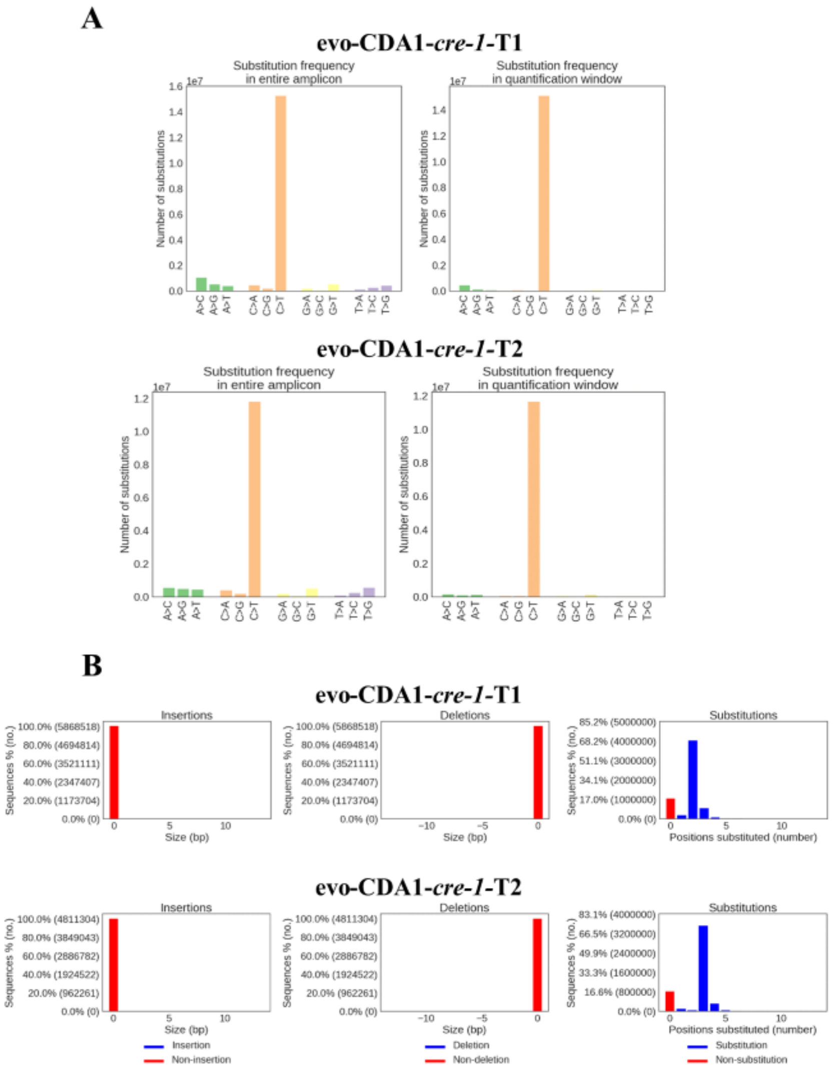

Fig. S4 Comparison of off-target between two sgRNAs of Mtevo-CDA1. (A) Two sgRNAs (sgRNA-*cre*-*1*-T1 and sgRNA-*cre*-*1*-T2) and Mtevo-CDA1 substitution frequency in entire amplicon and quantification window. The vertical coordinate shows the number of substitutions detected in the total sequencing data, and the horizontal coordinate shows the type of base substitution. The left panel shows the entire amplicon, and the right panel shows the target window. (B) Insertion, deletion and substitution locations distribution in two sgRNAs (sgRNA-*cre*-*1*-T1 and sgRNA-cre-1-T2) of Mtevo-CDA1.

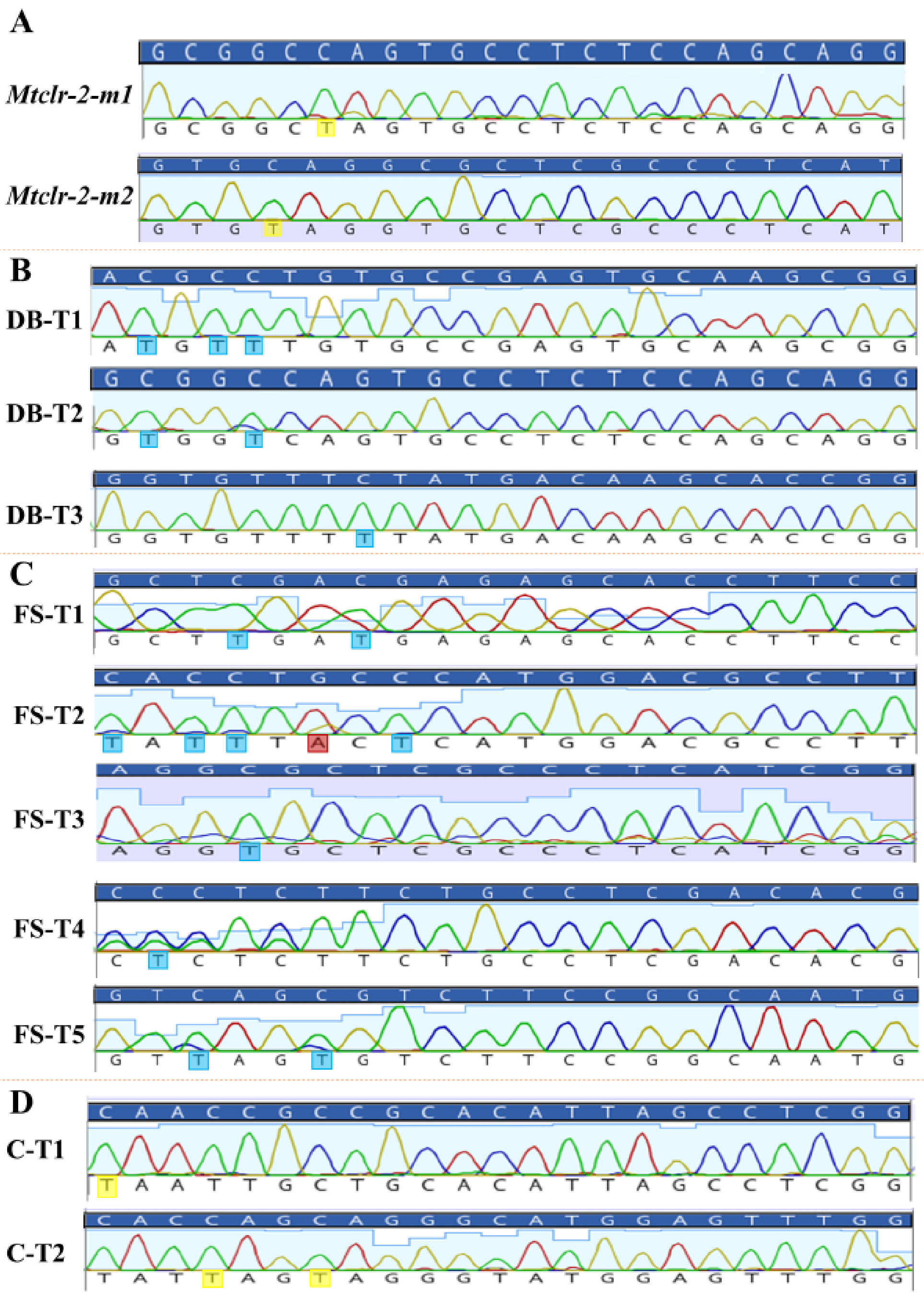

Fig. S5 Sequencing peak map of *Mtclr-2* gene mutant strains.

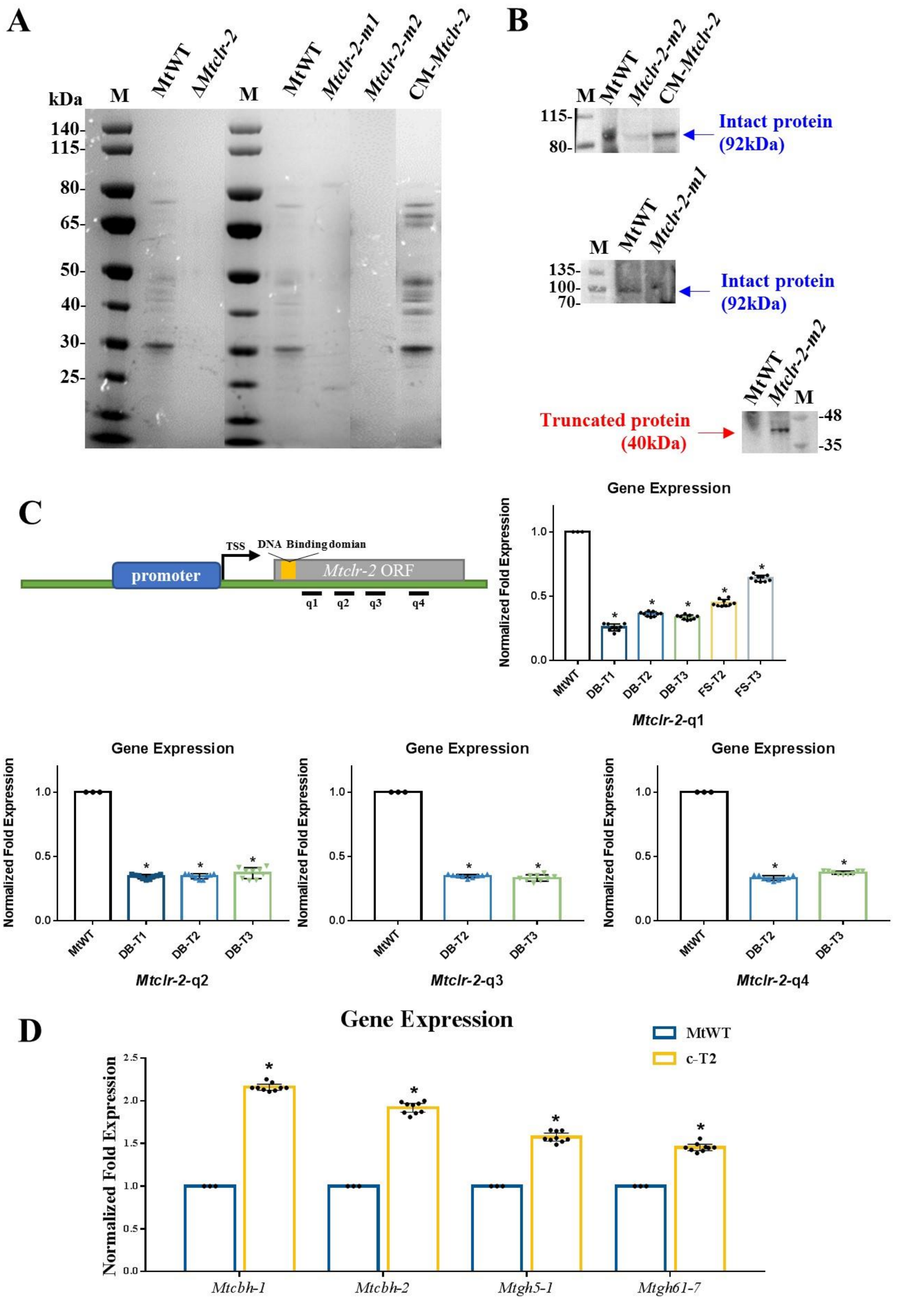

Fig. S6 Assays of the secreted protein concentration, western blotting and gene expression level in the MtCLR-2 mutants. (A) SDS-PAGE analysis of proteins secreted by Δ*Mtclr-2*, *Mtclr-2-m1*, *Mtclr-2-m1*, CM-*Mtclr-2* and MtWT strains after 4 days on 2% Avicel medium. (B) Western blotting

of intracellular protein from *Mtclr-2-m1* and *Mtclr-2-m2* and MtWT strains after a 4-h induction on 2% Avicel medium and probed with rabbit polyclonal antiserum. (C) Gene expression of *Mtclr-2* gene from the selected strains after a 4-h induction on 2% Avicel medium by using four pairs of the qPCR primers at different positions redesigned considering the gene truncation. (D) Gene expression levels of the major four MtCLR-2 target genes from the selected strains after a 4-h induction on 2% Avicel medium. Each black dot represents individual transformant. Bars marked by asterisks in each group differ significantly from the unmarked bars (Tukey's HSD, *$P < 0.05$). Error bars indicate the SD from multiple replicates.

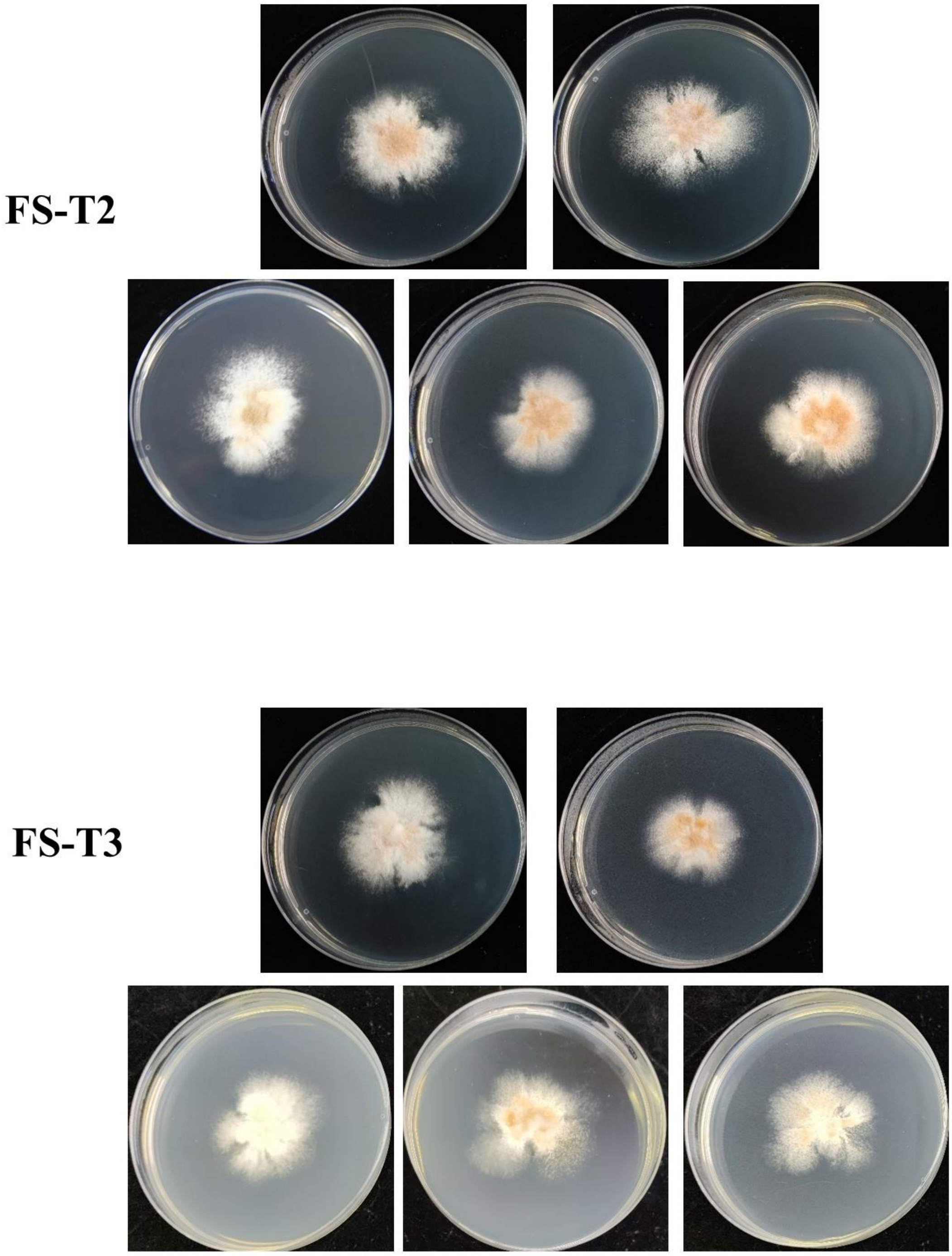

Fig. S7 Colony growth and sporulation of mutant strains (FS-T2 and FS-T3) on minimal medium plates after 4 days of culture.

Table S1 All the primer sequences used in this study.

Primer	Primer Sequence (5' - 3')
xcas9F_D10A_BE4	AGCTCCGGCGGTTCCGATAAGAAGTACTCCATCGGCCTCGCCATCGGCACCAACTCCGTC
xcas9F_D10A_Gam	AGCTCCGGCGGCAGCGATAAGAAGTACTCCATCGGCCTCGCCATCGGCACCAACTCCGTC
xcas9F_D10A_CDA1	TCCAGCGGCGGTTCCGATAAGAAGTACTCCATCGGCCTCGCCATCGGCACCAACTCCGTC
xcas9F_D10A_ABE	AGCTCTGGCGGCTCCGATAAGAAGTACTCCATCGGCCTCGCCATCGGCACCAACTCCGTC
xcas9_3.7up_R	GCTCATCGACGACCTTGACGGTCTGGAGGA
xcas9_3.7down_F	TATCGCCAACCTCGCCGGCTCCCCTGCCAT
xncas9_R_CBE	GTCGCCGCCGAGCTGGGAGAGATCGATGCGGGTTT
Gam_R_D10A	GACGGAGTTGGTGCCGATGGCGAGGCCGATGGAGTACTTCTTATCGCTGCCGCCGGAGCT
BE4max_R_D10A	GACGGAGTTGGTGCCGATGGCGAGGCCGATGGAGTACTTCTTATCGGAACCGCCGGAGCT
CDA1max_R_D10A	GACGGAGTTGGTGCCGATGGCGAGGCCGATGGAGTACTTCTTATCGGAACCGCCGCTGGA
All_CBE_F	TTTGCAGTTTTCATTAAGCGCAAGTCGCCAGTTTCGTTCTTCAGAATG
2UGI_F	GGCCTCTACGAAACCCGCATCGATCTCTCCCAGCTCGGCGGCGACTCGGGCGGTA
2UGI_R	TATCCAGATTCGTCAAGCTGTTTGATGATTTCAGTAACGTTAAGTTTATCATCACTCA
UGI_F_new	GGCCTCTACGAAACCCGCATCGATCTCTCCCAGCTCGGCGGCGACTCGGGCGGTAGCGGTGGTTCGGGCGGCAGCACGAACCTCAGCGACATTATCGAGAA
UGIBarT_F	GGCCTCTACGAAACCCGCATCGATCTCTCCCAGCT
UGIbarT_R_Hind3	AAGGCGATTAAGTTGGGTAACGCCAGGGTTTTCCCA
All_CBE_R	GCGGGTTTCGTAGAGGCCGGATCCGAATTCGATATCGTTTAAACACTAGTGACGGAGTTGGTGCCGATGGCGAGGCCGAT
All_UGIBarT_F	GCCATCGGCACCAACTCCGTCACTAGTGTTTAAACGATATCGAATTCGGATCCGGCCTCTACGAAACCCGCATCGATCTCT
nCas9_3.7_SF_3038	CCGAGTTCGTCTACGGTGATTACAA
Bar_SR_106	AGTTGACCGTGCTTGTCTCGATGTA
Ptef1_SF_194	CTTCGACCCCTCCTCAAATCTTCTT
pAN52_R2	AGGGGGATGTGCTGCAAGGCGATTA
TtrpC_SF_452	CAGGCTCCGGCGAAGAGAAGAATAG
ID_amdS_T1_F	TCATCGATACACTGGTTCTTGACAA
ID_amdS_T1_R	TCTGGACTTTCCATTCATCAGGGAT
ID_amdS_T2_F	CCTGGGAAGAACTGGCCGCTGATAA
ID_amdS_T2_R	TGGACACAAGATCTGCAGCGGAG

ID_amdS_T3_R	CACGAGTTCTTCCCTGACGC
ID_amdS_T3_R	CGGGACAGAGGTCTTGACGT
ID_clr2_T1_F	GTCACGACCTTCACACGACT
ID_clr2_T1_R	AGATGGGCGAGGTGTTGATG
ID_clr2_T2_F	GTCACGACCTTCACACGACT
ID_clr2_T2_R	AAGCCGCCAATCGAGGTATC
Deep_amds_F	TCTTCACTTCCTTCGTCGGC
Deep_amds_R	CCATCTTTGCATACGGCAGC
Deep_cre1_F	CTGAGATCTGGTGTTGGAGCA
Deep_cre1_R	GTAGGGACGGGGAAGTTCG
U6p2_F	AGGATCGGTGGAGTGAAGTTCGGAA
U6_clr2T1_R	GCTCTAAAACTGAAAGGTATGGAACATGCCGAGGAAAGAAAGAAAAGAAGAGGAG
U6_clr2T2_R	GCTCTAAAACGTGCTTGTCATAGAAACACCGAGGAAAGAAAGAAAAGAAGAGGAG
gRNA_R	AAAAAAGCACCGACTCGGTGCCACTT
gRNA_amdST1_F	TTCTTTCCTCGCCTCAATCCTGGGAAGAACGTTTTAGAGCTAGAAATAGCAAGTT
gRNA_amdST2_F	TTCTTTCCTCGCAAAGTCCAGACGCTGCCTGGTTTTAGAGCTAGAAATAGCAAGTT
gRNA_amdST3_F	TTCTTTCCTCCAAAGACCAGCTTCGAGTCAGTTTTAGAGCTAGAAATAGCAAGTT
gRNA_cre1T1_F	TTCTTTCCTCGCAACGCGCAAAGTCTGCAGGTTTTAGAGCTAGAAATAGCAAGTT
gRNA_cre1T2_F	TTCTTTCCTCACAAGCCGAAGGAGGAAACAGTTTTAGAGCTAGAAATAGCAAGTT
gRNA_clr2T1_F	TTCTTTCCTCGGCATGTTCCATACCTTTCAGTTTTAGAGCTAGAAATAGCAAGTT
gRNA_clr2T2_F	TTCTTTCCTCGGTGTTTCTATGACAAGCACGTTTTAGAGCTAGAAATAGCAAGTT
clr2_5F_Xba1	ACGAATTCGAGCTCGGTACCCGGGGATCCTCTAGACAGACTCACGAGTGTGGACGGCATT
clr2_5R	TGCTCCTTCAATATCAGTTAACGTCGAACATGCCGCCCTGTGTGGGCACT
Neo_F_clr2	AGTGCCCACACAGGGCGGCATGTTCGACGTTAACTGATATTGAAGGAGCA
Neo_R_clr2	CGAGGGTCTTGCGAGAGGGAATCACTCAGAAGAACTCGTCAAGAAGGCGA
clr2_3F	TCGCCTTCTTGACGAGTTCTTCTGAGTGATTCCCTCTCGCAAGACCCTCG
clr2_3R_Hind3	CGACGTTGTAAAACGACGGCCAGTGCCAAGCTTCGAGCATGGGGATGAAGACGTGGAT
puc118_F	GGCTCGTATGTTGTGTGGAATTGTG
puc118_R	GGTAACGCCAGGGTTTTCCCAGTCA
clr2_cm_F	TACTACGGTAAGGGAGGCCC

clr2_cm_R	TGGTGCTGTCCTGTGAATCC
ID_clr2_DB_F	TTCAAGGAGTTACCCCACGC
ID_clr2_DB_R	CTTCAGGATGGACCGGCATT
ID_clr2_C_F	GATTTGGACGGCATGTACTGG
ID_clr2_C_R	TGGACGTAGCCAAACTCCAT
ID_clr2_FS_F	CCCTGATCGACGCCTACTTC
ID_clr2_FS_R	CCTCGTACATGCGCCAGAC
Deep_clr2_FS_F1	CCCTGATCGACGCCTACTTC
Deep_clr2_FS_R1	GTAGTGGAGGTAGTAGCCGCC
Deep_clr2_FS_F2	ATCGTCGCGGCCGAGACCCGCCG
Deep_clr2_FS_R2	CGGCGGGTCTCGGCCGCGACGAT
Deep_clr2_FS_F3	ACCAGGCCGCCATGGTGCCCC
Deep_clr2_FS_R3	CGGCCTCGTACATGCGCCAGAC
Mtactin_RT_F	AACGCTCCTGCCTTCTAC
Mtactin_RT_R	GTAACACCATCACCAGAGTC
Mtclr2_RT_F1	ATGATGCCCAAGTCCGAGAG
Mtclr2_RT_R1	GTCAACCGAGAGCGAGAGG
Mtclr2_RT_F2	ATCCTGAAGCGGCTGTACC
Mtclr2_RT_R2	GTGTTGATGGGCGGTGATG
Mtclr2_RT_F3	CATCGCCGCCATGAAGTC
Mtclr2_RT_R3	TGATGTAGTGGAGGTAGTAGCC
Mtclr2_RT_F4	CGCCGTCTGGTTCCTCTAC
Mtclr2_RT_R4	GCGACCACTCCTCCATGATC
MtCBH1_RT_F	CTACAGCACCGACCGCTATG
MtCBH1_RT_R	CTTGAGGAACTGGGTGACGAC
MtCBH2_RT_F	CGGTGCCAATCCTCCCTATG
MtCBH2_RT_R	GCGTCGATGTAGCTCCTGTAG
MtEGL1_RT_F	ACATCATCACGGACACGAACG
MtEGL1_RT_R	GCCTGGTTGAGGTTGAGCAC
LPMO_RT_F	CATCCCGACCGAGTACATCAAC
LPMO _RT_R	CCTGCCATCTTCACCTGACTTG

Table S2 All gRNAs used in this study.

**Target Location**	**Protospacer Sequence(5' – 3')**	**Reference**
*amds*_T1	GCCTCAATCCTGGGAAGAAC	This study
*amds*_T2	GCAAAGTCCAGACGCTGCCTG	This study
*amds*_T3	CAAAGACCAGCTTCGAGTCA	This study
*cre1*_T1	GCAACGCGCAAAGTCTGCAG	This study
*cre1*_T2	ACAAGCCGAAGGAGGAAACA	This study
*clr2*_cas9_T1	GCGGCCAGTGCCTCTCCAGC	This study
*clr2*_cas9_T2	GGTGTTTCTATGACAAGCAC	This study
*clr2*_m_T1	GCGGCCAGTGCCTCTCCAGC	This study
*clr2*_m_T2	GTGCAGGCGCTCGCCCTCAT	This study
*clr2*_DB_T1	ACGCCTGTGCCGAGTGCAAG	This study
*clr2*_DB_T2	GCGGCCAGTGCCTCTCCAGC	This study
*clr2*_DB_T3	GGTGTTTCTATGACAAGCAC	This study
*clr2*_FS_T1	GCTCGACGAGAGCACCTTCC	This study
*clr2*_FS_T2	CACCTGCCCATGGACGCCTT	This study
*clr2*_FS_T3	GAGGCGCTCGCCCTCATCGG	This study
*clr2*_FS_T4	CCCTCTTCTGCCTCGACACG	This study
*clr2*_FS_T5	GTCAGCGTCTTCCGGCAATG	This study
*clr2*_C_T1	CAACCGCCGCACATTAGCCT	This study
*clr2*_C_T2	CACCAGCAGGGCATGGAGTT	This study

Note S1 Sequences of CBEs constructs.

evoBE4max

>Ptef1-NLS-evoAPOBEC1-xnCas9-3.7-linker-UGI-P2A-Bar-TtrpC

TCTGGCCATGTTCCGCATCTATCTACATCAGTTCAGTCTTTGCCCTGTTGGTCTTGCTAGCGGCGATGC
TATGAAAATCTATCAATTAATGGTGTCCTTTTTCGATGCGAGTACACTACTCATTTCGTTTCCGAATCG
CCATCTCTTCGACCATTGCTGAACTAATTCAATCTTAGGTAGTCTGCTGTTCCTTGTTTTCCATTTCCTT
GTCTTAAGTAAAACCAACTGGCACACCTCGAAACACGCTTGACGGATGGACAGTAGAATTGACCGTG
TACGTACATGTACCTTGACGTCCTCCGAGGTTCGACATCAGGGTTCGTCATAGGGAGTGAAACACCC
GCCATGATTCCGTAGCCGCGCGCGAAGATACGAAGCAGATATTTCACGGACATGGCGGAGATACTTG
TTTCCCGTACTAAGGTAGTCATGTCGGAGACATCTGAACGACAGAGCTGGCCAAGAGAACCGACCAG
TTGCCCCAGGACGATCTAGACAAAAAAAAAGAGAGATGAGTGGGCCACTTTTGCCACAACATCGAC
GGCCCTGCGACCGCCCCCAGGCAAACAAACAAACCGCCGAACAATAATACTTTTGTCATTTTAGGAG
GAGCGTTGTATGGATAAAAACAACATCTCGTTGCTGCAGAATGTGGACTTCAAACTTGCAGAAAATG
GGAGGCGGATTTGCATGATCGGAGGGTAGTTGACTCACGCCGCAGGCTGCAAATCCGTCCTCCATTA
TTCCATGAACAACTTCGTAAGGTTGGGCTGAGCGCCAATGCCTAACGGACCGGGGGCCACAGCGCAA
CGTCCCACTTAAAGGCCAGCGTGACATGCCAGTTCCATACCAAGTAGTGGCACCAGAGGCGGCCAAT
GCTCAGTAAGGGCAGGGAGGGAGGCTCAAACGATTGGCAAAAAGAGGGGCTTGCCAGTTCAGTTCC
CTGTGCGAGCGCGAGAGGGGCAGTTTCAAATCTGGAGGGGTGTGTTGCGCTGGTCTGAAGAGAAAG
AGAAGACTGTACTTAATAATTGTTCAAAGAGTCCATCATCGCGTTGCGGACTCCTCTAGCTGTATTTA
GAGCCCTATCATTACTTGTCGGGTGCGAATCAAAATACCGGGATGCAGCCCTCTGGCGATTTGCATG
CGGTTGTGGAGGAAGTGAAGCCTGAATCGCGGGGCTGGGCGGCAAAGCACGACGTGAAATTCCTGG
CGAAATTCGAGGGCTTGCCCCACCGTGGTTGAAGTTTTTGTGCTGCGTAACCCCACCAACCCGCCTTG
CCCCTCCCGCCTGCCCATAAAAACTTCGACCCCTCCTCAAATCTTCTTCGATTCTTCCTCTTCACTTCC
TTCGTCGGCATACCTGATTCAAGCAATCACCTGCCACTTTCAAGTGCGTATACCATCATCGATACACT
GGTTCTTGACAAGTACATCGTCTCTAACTTTCCTTTTTGCAGTTTTCATTAAGCGCAAGTCGCCAGTTT
CGTTCTTCAGAATGCCTCCAAGGAAACGCGCGAAGACCGAGGACGAGAAGCGCACCGCCGACGGCT
CCGAGTTCGAGTCCCCTAAGAAAAAGCGCAAGGTCTCCTCCAAGACCGGTCCTGTCGCCGTCGATCCT
ACTCTCCGCCGTCGCATCGAGCCTCACGAGTTCGAGGTGTTCTTCGATCCTCGCGAGCTGCGCAAAGA
AACCTGCCTCCTCTACGAGATCAACTGGGGCGGTCGCCATTCCATCTGGCGCCATACCAGCCAGAACA
CCAACAAGCACGTCGAGGTCAACTTCATCGAAAAGTTTACCACCGAGCGCTATTTCTGCCCTAACACT
CGCTGCTCTATTACCTGGTTCCTCAGCTGGTCCCCTTGCGGCGAGTGCTCCCGCGCCATCACCGAGTTC
CTTTCTCGCTACCCTAACGTCACCCTCTTCATCTATATCGCCCGCCTCTACCATCTCGCTAACCCTCGCA
ACCGCCAGGGCCTCCGCGATCTCATCTCCTCCGGCGTCACCATCCAGATCATGACCGAGCAAGAGTCC
GGCTACTGCTGGCATAACTTCGTCAACTACTCCCCATCCAACGAGTCCCATTGGCCACGCTACCCTCAT
CTCTGGGTCCGCCTGTACGTCCTCGAGCTGTACTGTATTATCCTCGGCCTGCCTCCATGCCTCAACATCC
TCCGCCGCAAGCAGTCCCAGCTCACCTCCTTCACTATCGCCCTCCAGTCCTGCCATTACCAGCGCCTGC
CACCTCATATCCTCTGGGCCACCGGCCTCAAGTCCGGCGGCTCCTCCGGTGGCTCCAGCGGCTCCGAG
ACTCCTGGCACCTCCGAGAGCGCTACCCCTGAGTCCAGCGGCGGCAGCTCCGGCGGTTCCGATAAGA
AGTACTCCATCGGCCTCGCCATCGGCACCAACTCCGTCGGCTGGGCCGTCATCACCGATGAGTACAA
GGTCCCTTCCAAGAAGTTCAAGGTCCTCGGCAACACCGATCGCCATTCCATCAAGAAGAACCTGATC
GGCGCCCTCCTGTTCGATTCCGGCGAAACCGCCGAGGCCACCCGCCTTAAACGCACCGCCCGTCGCC
GCTACACCCGCCGCAAGAACCGCATCTGCTACCTCCAAGAAATCTTCTCCAACGAGATGGCCAAGGT
CGATGATAGCTTCTTCCACCGCCTCGAAGAGTCCTTCCTGGTCGAAGAGGATAAGAAGCACGAGCGC

CATCCTATCTTCGGCAACATCGTCGATGAGGTCGCCTACCATGAGAAGTACCCTACCATCTACCATCT
CCGCAAGAAGCTCGTCGATTCCACCGATAAGGCCGATCTCCGCCTCATCTACCTCGCCCTCGCCCATA
TGATCAAGTTCCGCGGCCATTTCCTCATCGAGGGCGATCTCAACCCTGATAACTCCGATGTCGATAAG
CTGTTCATCCAGCTCGTCCAGACCTACAACCAGCTGTTCGAGGAAAACCCTATCAACGCCTCCGGCGT
CGATGCCAAGGCCATCCTCTCCGCTCGCCTCTCCAAGTCTCGCCGCCTTGAGAACCTTATCGCCCAGC
TCCCTGGCGAGAAGAAGAACGGCCTCTTCGGCAACCTGATCGCCCTCTCCCTCGGCCTCACCCCTAAC
TTCAAGTCCAACTTCGATCTCGCCGAGGATACCAAGCTCCAGCTCTCCAAGGATACCTACGATGATG
ATCTCGATAACCTCCTCGCCCAGATCGGCGATCAGTACGCCGATCTGTTCCTCGCCGCCAAGAACCTC
TCCGATGCCATCCTCCTCTCCGACATCCTCCGCGTCAACACCGAGATCACCAAGGCCCCTCTGTCCGC
CTCCATGATCAAGCTCTACGATGAGCATCATCAGGACCTCACCCTGCTCAAGGCCCTCGTCCGCCAGC
AGCTCCCTGAGAAGTACAAAGAGATTTTCTTCGATCAGTCCAAGAACGGCTACGCCGGCTACATCGA
TGGCGGCGCTTCCCAAGAAGAGTTCTACAAGTTCATCAAGCCTATCCTTGAGAAGATGGATGGCACC
GAGGAACTCCTCGTCAAGCTCAACCGCGAGGACCTCCTCCGCAAGCAGCGCACCTTCGATAACGGCA
TCATCCCTCATCAAATCCATCTCGGCGAGCTGCATGCCATCTTGCGCCGCCAAGAGGATTTCTACCCA
TTCCTCAAGGATAACCGCGAGAAGATCGAAAAGATTCTCACCTTCCGCATCCCTTACTACGTCGGCCC
TCTCGCTCGCGGCAACTCCCGCTTCGCCTGGATGACCCGCAAGTCCGAGGAAACCATCACCCCTTGG
AACTTCGAGAAAGTCGTCGATAAGGGCGCCTCCGCCCAGTCCTTCATCGAGCGCATGACCAACTTCG
ATAAGAACCTCCCTAACGAGAAGGTCCTCCCTAAGCACTCCCTGCTCTACGAGTACTTCACCGTCTAC
AACGAGCTGACCAAGGTCAAGTACGTCACCGAGGGTATGCGCAAGCCTGCCTTCCTGTCCGGCGATC
AGAAGAAGGCCATCGTCGATCTGCTGTTCAAGACCAACCGCAAGGTCACCGTCAAGCAGCTCAAAG
AGGATTACTTCAAGAAAATCGAGTGCTTCGATTCCGTCGAGATCAGCGGCGTCGAGGACCGCTTCAA
CGCCTCCCTCGGAACCTACCATGATCTCCTCAAGATTATCAAGGATAAGGATTTCCTCGACAACGAG
GAAAACGAGGACATCCTTGAGGACATCGTCCTCACCCTCACCCTCTTCGAGGACCGCGAAATGATCG
AGGAACGCCTCAAGACCTACGCCCATCTCTTCGATGATAAGGTCATGAAGCAGCTCAAGCGCCGTCG
CTACACCGGCTGGGGTCGCCTCTCCCGCAAGCTCATCAACGGCATCCGCGATAAGCAGTCCGGCAAG
ACTATCCTCGATTTCCTCAAGTCCGATGGCTTCGCCAACCGCAACTTCATTCAGCTCATCCATGATGA
TTCCCTCACCTTCAAAGAGGACATCCAGAAGGCCCAGGTCAGCGGCCAGGGCGATTCCCTCCATGAG
CATATCGCCAACCTCGCCGGCTCCCCTGCCATCAAGAAGGGCATCCTCCAGACCGTCAAGGTCGTCG
ATGAGCTGGTCAAGGTCATGGGCCGCCATAAGCCTGAGAACATCGTCATCGAGATGGCCCGCGAGA
ACCAGACCACCCAGAAGGGCCAGAAGAACTCCCGCGAGCGCATGAAGCGCATCGAGGAAGGCATCA
AAGAGCTGGGCAGCCAAATCCTCAAAGAGCATCCTGTCGAGAACACCCAGCTCCAGAACGAGAAGC
TCTACCTCTACTACCTCCAGAACGGCCGCGATATGTACGTCGATCAAGAGCTGGACATCAACCGCCT
CTCCGATTACGATGTCGATCATATCGTCCCTCAGTCCTTCCTGAAGGATGATTCCATCGATAACAAGG
TCCTCACCCGCTCCGATAAGAACCGCGGCAAGTCCGATAACGTCCCTTCCGAAGAGGTCGTCAAGAA
GATGAAGAACTACTGGCGCCAGCTCCTCAACGCCAAGCTCATCACCCAGCGCAAGTTCGATAACCTC
ACCAAGGCCGAGCGCGGTGGCCTCTCCGAGCTGGATAAGGCCGGCTTCATCAAGCGCCAGCTCGTCG
AAACCCGCCAGATCACCAAGCACGTCGCCCAAATCCTCGATTCCCGCATGAACACCAAGTACGATGA
GAACGATAAGCTCATCCGCGAAGTCAAGGTCATCACCCTCAAGTCCAAGCTCGTCAGCGATTTCCGC
AAGGATTTCCAGTTCTACAAGGTCCGCGAGATCAACAACTACCATCATGCCCATGATGCCTACCTCA
ACGCCGTCGTCGGCACCGCCCTCATCAAGAAGTACCCCAAGCTCGAATCCGAGTTCGTCTACGGTGA
TTACAAGGTCTACGATGTCCGCAAGATGATCGCCAAGTCCGAGCAAGAGATCGGCAAGGCTACCGCC
AAGTACTTCTTCTACTCCAACATCATGAATTTCTTCAAGACCGAAATCACCCTCGCCAACGGCGAAAT
CCGCAAGCGCCCTCTCATCGAGACTAACGGCGAGACTGGCGAGATCGTCTGGGATAAGGGCCGCGAT
TTCGCCACCGTCCGCAAGGTCCTCTCCATGCCTCAGGTCAACATCGTCAAGAAAACCGAGGTCCAGA

CCGGCGGCTTCTCCAAAGAGTCCATCCTCCCCAAGCGCAACTCCGATAAGCTGATCGCCCGCAAGAA
GGATTGGGACCCTAAGAAGTACGGCGGCTTCGATTCCCCTACCGTCGCCTACTCCGTCCTCGTCGTCG
CCAAGGTCGAGAAGGGCAAGTCCAAGAAGCTCAAGTCCGTCAAAGAGCTGCTCGGCATCACTATTAT
GGAACGCTCCAGCTTCGAGAAGAACCCTATCGATTTCCTTGAGGCCAAGGGCTACAAAGAGGTCAAG
AAGGACCTCATCATCAAGCTCCCCAAGTACTCCCTGTTCGAGCTTGAGAACGGCCGCAAGCGCATGC
TCGCCTCCGCCGGTGTGCTTCAGAAGGGCAACGAGCTGGCCCTGCCTTCCAAGTACGTCAACTTCCTC
TACCTCGCCTCCCATTACGAGAAGCTCAAGGGCTCCCCTGAGGATAACGAGCAGAAGCAGCTGTTCG
TCGAGCAGCATAAGCACTACCTCGATGAGATCATCGAGCAGATCAGCGAGTTCTCCAAGCGCGTCAT
CCTCGCCGATGCCAACCTCGATAAGGTCCTGTCCGCCTACAACAAGCACCGCGATAAGCCTATCCGC
GAGCAGGCCGAGAACATCATCCATCTCTTCACCCTCACCAACCTCGGTGCCCCTGCCGCCTTCAAGTA
CTTCGATACCACCATCGATCGCAAGCGCTACACCTCCACCAAAGAGGTCCTGGACGCCACCCTCATC
CATCAGTCCATCACCGGCCTCTACGAAACCCGCATCGATCTCTCCCAGCTCGGCGGCGACTCGGGCG
GTAGCGGTGGTTCGGGCGGCAGCACGAACCTCAGCGACATTATCGAGAAGGAGACGGGCAAGCAGCT
CGTCATCCAAGAGTCGATCCTCATGCTGCCCGAGGAGGTCGAAGAGGTCATCGGCAACAAGCCCGAG
TCGGACATCCTCGTCCATACCGCCTACGACGAGAGCACCGATGAAAACGTCATGCTGCTGACCAGCGA
CGCCCCCGAGTATAAGCCTTGGGCTCTCGTGATTCAAGACTCGAATGGCGAGAACAAGATCAAGATGC
TCAGCGGTGGCAGCGGCGGTTCGGGTGGTTCGACCAACCTCAGCGACATCATCGAAAAGGAGACCGG
CAAGCAGCTCGTCATCCAAGAGAGCATTCTCATGCTGCCCGAGGAGGTGGAGGAGGTCATTGGCAAC
AAGCCCGAGAGCGACATTCTCGTGCACACCGCCTACGATGAGAGCACCGACGAGAACGTGATGCTGC
TGACCAGCGATGCCCCCGAGTACAAGCCTTGGGCTCTGGTCATTCAAGACAGCAACGGCGAGAACAA
AATCAAGATGCTGAGCGGCGGTAGCGACCCCAAGAAGAAACGCAAGGTTGATCCGAAGAAGAAGCG
GAAGGTTCCTCCGAGGAAACGTGCCAAAACAGAAGATGAGGGAAGCGGAGCTACTAACTTCAGCCT
GCTGAAGCAGGCTGGAGACGTGGAGGAGAACCCTGGACCTATGAGCCCAGAACGACGCCCGGCCGA
CATCCGCCGTGCCACCGAGGCGGACATGCCGGCGGTCTGCACCATCGTCAACCACTACATCGAGACA
AGCACGGTCAACTTCCGTACCGAGCCGCAGGAACCGCAGGAGTGGACGGACGACCTCGTCCGTCTGC
GGGAGCGCTATCCCTGGCTCGTCGCCGAGGTGGACGGCGAGGTCGCCGGCATCGCCTACGCGGGCCC
CTGGAAGGCACGCAACGCCTACGACTGGACGGCCGAGTCGACCGTGTACGTCTCCCCCCGCCACCAG
CGGACGGGACTGGGCTCCACGCTCTACACCCACCTGCTGAAGTCCCTGGAGGCACAGGGCTTCAAGA
GCGTGGTCGCTGTCATCGGGCTGCCCAACGACCCGAGCGTGCGCATGCACGAGGCGCTCGGATATGC
CCCCCGCGGCATGCTGCGGGCGGCCGGCTTCAAGCACGGGAACTGGCATGACGTGGGTTTCTGGCAG
CTGGACTTCAGCCTGCCGGTACCGCCCCGTCCGGTCCTGCCCGTCACCGAGATTTGATGAACTTAACG
TTACTGAAATCATCAAACAGCTTGACGAATCTGGATATAAGATCGTTGGTGTCGATGTCAGCTCCGGAG
TTGAGACAAATGGTGTTCAGGATCTCGATAAGATACGTTCATTTGTCCAAGCAGCAAAGAGTGCCTTCT
AGTGATTTAATAGCTCCATGTCAACAAGAATAAAACGCGTTTTCGGGTTTACCTCTTCCAGATACAGCT
CATCTGCAATGCATTAATGCATTGACTGCAACCTAGTAACGCCTTTCAGGCTCCGGCGAAGAGAAGAA
TAGCTTAGCAGAGCTATTTTCATTTTCGGGAGACGAGATCAAGCAGATCAACGGTCGTCAAGAGACCT
ACGAGACTGAGGAATCCGCTCTTGGCTCCACGCGACTATATATTTGTCTCTAATTGTACTTTGACATGCT
CCTCTTCTTTACTCTGATAGCTTGACTATGAAAATTCCGTCACCAGCTCCTGGGTTCGCAAAGATAATTG
CATGTTTCTTCCTTGAACTCTCAAGCCTACAGGACACACATTCATCGTAGGTATAAACCTCGAAATCAT
TTCCTACTAAGATGGTATACAATAGTAACCATGCATGGTTGCCTAGTGAATGCTCCGTAACACCCAATAC
GCCGGCCGAAACTTTTTTACAACTCTCCTATGAGTCGTTTACCCAGAATGCACAGGTACACTTGTTTAG
AGGTAATCCTTCTT

Gam-evoBE4max

>Ptef1-NLS-Gam-evoAPOBEC1-xnCas9-3.7-linker-UGI-P2A-Bar-TtrpC

TCTGGCCATGTTCCGCATCTATCTACATCAGTTCAGTCTTTGCCCTGTTGGTCTTGCTAGCGGCGATGC
TATGAAAATCTATCAATTAATGGTGTCCTTTTTCGATGCGAGTACACTACTCATTTCGTTTCCGAATCG
CCATCTCTTCGACCATTGCTGAACTAATTCAATCTTAGGTAGTCTGCTGTTCCTTGTTTTCCATTTCCTT
GTCTTAAGTAAAACCAACTGGCACACCTCGAAACACGCTTGACGGATGGACAGTAGAATTGACCGTG
TACGTACATGTACCTTGACGTCCTCCGAGGTTCGACATCAGGGTTCGTCATAGGGAGTGAAACACCC
GCCATGATTCCGTAGCCGCGCGAAGATACGAAGCAGATATTTCACGGACATGGCGGAGATACTTG
TTTCCCGTACTAAGGTAGTCATGTCGGAGACATCTGAACGACAGAGCTGGCCAAGAGAACCGACCAG
TTGCCCCAGGACGATCTAGACAAAAAAAAAGAGAGATGAGTGGGCCACTTTTGCCACAACATCGAC
GGCCCTGCGACCGCCCCCAGGCAAACAAACAAACCGCCGAACAATAATACTTTTGTCATTTTAGGAG
GAGCGTTGTATGGATAAAAACAACATCTCGTTGCTGCAGAATGTGGACTTCAAACTTGCAGAAAATG
GGAGGCGGATTTGCATGATCGGAGGGTAGTTGACTCACGCCGCAGGCTGCAAATCCGTCCTCCATTA
TTCCATGAACAACTTCGTAAGGTTGGGCTGAGCGCCAATGCCTAACGGACCGGGGGCCACAGCGCAA
CGTCCCACTTAAAGGCCAGCGTGACATGCCAGTTCCATACCAAGTAGTGGCACCAGAGGCGGCCAAT
GCTCAGTAAGGGCAGGGAGGGAGGCTCAAACGATTGGCAAAAAGAGGGGCTTGCCAGTTCAGTTCC
CTGTGCGAGCGCGAGAGGGGCAGTTTCAAATCTGGAGGGGTGTGTTGCGCTGGTCTGAAGAGAAAG
AGAAGACTGTACTTAATAATTGTTCAAAGAGTCCATCATCGCGTTGCGGACTCCTCTAGCTGTATTTA
GAGCCCTATCATTACTTGTCGGGTGCGAATCAAAATACCGGGATGCAGCCCTCTGGCGATTTGCATG
CGGTTGTGGAGGAAGTGAAGCCTGAATCGCGGGGCTGGGCGGCAAAGCACGACGTGAAATTCCTGG
CGAAATTCGAGGGCTTGCCCCACCGTGGTTGAAGTTTTTGTGCTGCGTAACCCCACCAACCCGCCTTG
CCCCTCCCGCCTGCCCATAAAAACTTCGACCCCTCCTCAAATCTTCTTCGATTCTTCCTCTTCACTTCC
TTCGTCGGCATACCTGATTCAAGCAATCACCTGCCACTTTCAAGTGCGTATACCATCATCGATACACT
GGTTCTTGACAAGTACATCGTCTCTAACTTTCCTTTTTGCAGTTTTCATTAAGCGCAAGTCGCCAGTTT
CGTTCTTCAGAATGCCTCCAAGGAAACGCGCGAAGACCGAGGACGAGAAGCGCACCGCCGACGGCT
CCGAGTTCGAGTCCCCTAAGAAAAAGCGCAAGGTCGCCAAGCCTGCCAAGCGCATCAAGTCCGCCGC
TGCCGCTTACGTCCCTCAGAACCGCGACGCCGTCATCACCGATATCAAGCGGATCGGCGATCTCCAGC
GCGAGGCCAGCCGCCTCGAAACCGAGATGAACGATGCTATCGCCGAGATCACCGAGAAGTTCGCCGC
TCGGATCGCCCCTATCAAGACCGATATCGAAACCCTCTCCAAGGGCGTCCAAGGCTGGTGCGAGGCCA
ACCGGGATGAGCTGACCAACGGCGGCAAGGTCAAGACCGCCAACCTCGTGACCGGTGATGTCTCCTG
GCGCGTGCGCCCTCCTTCTGTCTCCATCCGTGGCATGGATGCCGTCATGGAAACCCTCGAGCGCCTCG
GCCTCCAGCGCTTCATCCGCACCAAGCAAGAGATCAACAAAGAGGCCATCCTCCTCGAGCCTAAGGC
CGTCGCCGGCGTCGCTGGCATCACCGTCAAGTCCGGCATCGAGGATTTCAGCATCATCCCTTTCGAGC
AAGAGGCCGGCATCTCCGGCTCCGAGACTCCTGGCACCTCCGAGAGCGCTACCCCTGAGTCCTCCTCC
AAGACCGGTCCTGTCGCCGTCGATCCTACTCTCCGCCGTCGCATCGAGCCTCATGAGTTCGAGGTGTT
CTTCGATCCTCGCGAGCTGCGCAAAGAAACCTGCCTCCTCTACGAGATCAACTGGGGCGGTCGCCATT
CCATCTGGCGCCATACCAGCCAGAACACCAACAAGCACGTCGAGGTCAACTTCATCGAAAAGTTTACC
ACCGAGCGCTATTTCTGCCCTAACACTCGCTGCTCTATTACCTGGTTCCTCAGCTGGTCCCCTTGCGGC
GAGTGCTCCCGCGCCATCACCGAGTTCCTTTCTCGCTACCCTAACGTCACCCTCTTCATCTATATCGCCC
GCCTCTACCATCTCGCTAACCCTCGCAACCGCCAGGGCCTCCGCGATCTCATCTCCTCCGGCGTCACCA
TCCAGATCATGACCGAGCAAGAATCCGGCTACTGCTGGCATAACTTCGTCAACTACTCCCCATCCAACG
AGTCCCATTGGCCACGCTACCCTCATCTCTGGGTCCGCCTGTACGTCCTCGAGCTGTACTGTATTATCCT
CGGCCTGCCTCCATGCCTCAACATCCTCCGCCGCAAGCAGTCCCAGCTCACCTCCTTCACTATCGCCCT
CCAGTCCTGCCATTACCAGCGCCTGCCACCTCATATCCTCTGGGCCACCGGCCTGAAGTCCGGCGGCT
CCTCCGGTGGCTCCAGCGGCTCTGAAACCCCAGGCACCAGCGAGTCTGCCACTCCTGAGTCCTCTGG

CGGCAGCTCCGGCGGCAGCGATAAGAAGTACTCCATCGGCCTCGCCATCGGCACCAACTCCGTCGGC
TGGGCCGTCATCACCGATGAGTACAAGGTCCCTTCCAAGAAGTTCAAGGTCCTCGGCAACACCGATC
GCCATTCCATCAAGAAGAACCTGATCGGCGCCCTCCTGTTCGATTCCGGCGAAACCGCCGAGGCCAC
CCGCCTTAAACGCACCGCCCGTCGCCGCTACACCCGCCGCAAGAACCGCATCTGCTACCTCCAAGAA
ATCTTCTCCAACGAGATGGCCAAGGTCGATGATAGCTTCTTCCACCGCCTCGAAGAGTCCTTCCTGGT
CGAAGAGGATAAGAAGCACGAGCGCCATCCTATCTTCGGCAACATCGTCGATGAGGTCGCCTACCAT
GAGAAGTACCCTACCATCTACCATCTCCGCAAGAAGCTCGTCGATTCCACCGATAAGGCCGATCTCC
GCCTCATCTACCTCGCCCTCGCCCATATGATCAAGTTCCGCGGCCATTTCCTCATCGAGGGCGATCTC
AACCCTGATAACTCCGATGTCGATAAGCTGTTCATCCAGCTCGTCCAGACCTACAACCAGCTGTTCGA
GGAAAACCCTATCAACGCCTCCGGCGTCGATGCCAAGGCCATCCTCTCCGCTCGCCTCTCCAAGTCTC
GCCGCCTTGAGAACCTTATCGCCCAGCTCCCTGGCGAGAAGAAGAACGGCCTCTTCGGCAACCTGAT
CGCCCTCTCCCTCGGCCTCACCCCTAACTTCAAGTCCAACTTCGATCTCGCCGAGGATACCAAGCTCC
AGCTCTCCAAGGATACCTACGATGATGATCTCGATAACCTCCTCGCCCAGATCGGCGATCAGTACGC
CGATCTGTTCCTCGCCGCCAAGAACCTCTCCGATGCCATCCTCCTCTCCGACATCCTCCGCGTCAACA
CCGAGATCACCAAGGCCCCTCTGTCCGCCTCCATGATCAAGCTCTACGATGAGCATCATCAGGACCT
CACCCTGCTCAAGGCCCTCGTCCGCCAGCAGCTCCCTGAGAAGTACAAAGAGATTTTCTTCGATCAGT
CCAAGAACGGCTACGCCGGCTACATCGATGGCGGCGCTTCCCAAGAAGAGTTCTACAAGTTCATCAA
GCCTATCCTTGAGAAGATGGATGGCACCGAGGAACTCCTCGTCAAGCTCAACCGCGAGGACCTCCTC
CGCAAGCAGCGCACCTTCGATAACGGCATCATCCCTCATCAAATCCATCTCGGCGAGCTGCATGCCA
TCTTGCGCCGCCAAGAGGATTTCTACCCATTCCTCAAGGATAACCGCGAGAAGATCGAAAAGATTCT
CACCTTCCGCATCCCTTACTACGTCGGCCCTCTCGCTCGCGGCAACTCCCGCTTCGCCTGGATGACCC
GCAAGTCCGAGGAAACCATCACCCCTTGGAACTTCGAGAAAGTCGTCGATAAGGGCGCCTCCGCCCA
GTCCTTCATCGAGCGCATGACCAACTTCGATAAGAACCTCCCTAACGAGAAGGTCCTCCCTAAGCAC
TCCCTGCTCTACGAGTACTTCACCGTCTACAACGAGCTGACCAAGGTCAAGTACGTCACCGAGGGTA
TGCGCAAGCCTGCCTTCCTGTCCGGCGATCAGAAGAAGGCCATCGTCGATCTGCTGTTCAAGACCAA
CCGCAAGGTCACCGTCAAGCAGCTCAAAGAGGATTACTTCAAGAAAATCGAGTGCTTCGATTCCGTC
GAGATCAGCGGCGTCGAGGACCGCTTCAACGCCTCCCTCGGAACCTACCATGATCTCCTCAAGATTA
TCAAGGATAAGGATTTCCTCGACAACGAGGAAAACGAGGACATCCTTGAGGACATCGTCCTCACCCT
CACCCTCTTCGAGGACCGCGAAATGATCGAGGAACGCCTCAAGACCTACGCCCATCTCTTCGATGAT
AAGGTCATGAAGCAGCTCAAGCGCCGTCGCTACACCGGCTGGGGTCGCCTCTCCCGCAAGCTCATCA
ACGGCATCCGCGATAAGCAGTCCGGCAAGACTATCCTCGATTTCCTCAAGTCCGATGGCTTCGCCAA
CCGCAACTTCATTCAGCTCATCCATGATGATTCCCTCACCTTCAAAGAGGACATCCAGAAGGCCCAG
GTCAGCGGCCAGGGCGATTCCCTCCATGAGCATATCGCCAACCTCGCCGGCTCCCCTGCCATCAAGA
AGGGCATCCTCCAGACCGTCAAGGTCGTCGATGAGCTGGTCAAGGTCATGGGCCGCCATAAGCCTGA
GAACATCGTCATCGAGATGGCCCGCGAGAACCAGACCACCCAGAAGGGCCAGAAGAACTCCCGCGA
GCGCATGAAGCGCATCGAGGAAGGCATCAAAGAGCTGGGCAGCCAAATCCTCAAAGAGCATCCTGT
CGAGAACACCCAGCTCCAGAACGAGAAGCTCTACCTCTACTACCTCCAGAACGGCCGCGATATGTAC
GTCGATCAAGAGCTGGACATCAACCGCCTCTCCGATTACGATGTCGATCATATCGTCCCTCAGTCCTT
CCTGAAGGATGATTCCATCGATAACAAGGTCCTCACCCGCTCCGATAAGAACCGCGGCAAGTCCGAT
AACGTCCCTTCCGAAGAGGTCGTCAAGAAGATGAAGAACTACTGGCGCCAGCTCCTCAACGCCAAGC
TCATCACCCAGCGCAAGTTCGATAACCTCACCAAGGCCGAGCGCGGTGGCCTCTCCGAGCTGGATAA
GGCCGGCTTCATCAAGCGCCAGCTCGTCGAAACCCGCCAGATCACCAAGCACGTCGCCCAAATCCTC
GATTCCCGCATGAACACCAAGTACGATGAGAACGATAAGCTCATCCGCGAAGTCAAGGTCATCACCC
TCAAGTCCAAGCTCGTCAGCGATTTCCGCAAGGATTTCCAGTTCTACAAGGTCCGCGAGATCAACAA

CTACCATCATGCCCATGATGCCTACCTCAACGCCGTCGTCGGCACCGCCCTCATCAAGAAGTACCCCA
AGCTCGAATCCGAGTTCGTCTACGGTGATTACAAGGTCTACGATGTCCGCAAGATGATCGCCAAGTC
CGAGCAAGAGATCGGCAAGGCTACCGCCAAGTACTTCTTCTACTCCAACATCATGAATTTCTTCAAG
ACCGAAATCACCCTCGCCAACGGCGAAATCCGCAAGCGCCCTCTCATCGAGACTAACGGCGAGACTG
GCGAGATCGTCTGGGATAAGGGCCGCGATTTCGCCACCGTCCGCAAGGTCCTCTCCATGCCTCAGGT
CAACATCGTCAAGAAAACCGAGGTCCAGACCGGCGGCTTCTCCAAAGAGTCCATCCTCCCCAAGCGC
AACTCCGATAAGCTGATCGCCCGCAAGAAGGATTGGGACCCTAAGAAGTACGGCGGCTTCGATTCCC
CTACCGTCGCCTACTCCGTCCTCGTCGTCGCCAAGGTCGAGAAGGGCAAGTCCAAGAAGCTCAAGTC
CGTCAAAGAGCTGCTCGGCATCACTATTATGGAACGCTCCAGCTTCGAGAAGAACCCTATCGATTTC
CTTGAGGCCAAGGGCTACAAAGAGGTCAAGAAGGACCTCATCATCAAGCTCCCCAAGTACTCCCTGT
TCGAGCTTGAGAACGGCCGCAAGCGCATGCTCGCCTCCGCCGGTGTGCTTCAGAAGGGCAACGAGCT
GGCCCTGCCTTCCAAGTACGTCAACTTCCTCTACCTCGCCTCCCATTACGAGAAGCTCAAGGGCTCCC
CTGAGGATAACGAGCAGAAGCAGCTGTTCGTCGAGCAGCATAAGCACTACCTCGATGAGATCATCGA
GCAGATCAGCGAGTTCTCCAAGCGCGTCATCCTCGCCGATGCCAACCTCGATAAGGTCCTGTCCGCCT
ACAACAAGCACCGCGATAAGCCTATCCGCGAGCAGGCCGAGAACATCATCCATCTCTTCACCCTCAC
CAACCTCGGTGCCCCTGCCGCCTTCAAGTACTTCGATACCACCATCGATCGCAAGCGCTACACCTCCA
CCAAAGAGGTCCTGGACGCCACCCTCATCCATCAGTCCATCACCGGCCTCTACGAAACCCGCATCGA
TCTCTCCCAGCTCGGCGGCGACTCGGGCGGTAGCGGTGGTTCGGGCGGCAGCACGAACCTCAGCGAC
ATTATCGAGAAGGAGACGGGCAAGCAGCTCGTCATCCAAGAGTCGATCCTCATGCTGCCCGAGGAG
TCGAAGAGGTCATCGGCAACAAGCCCGAGTCGGACATCCTCGTCCATACCGCCTACGACGAGAGCAC
CGATGAAAACGTCATGCTGCTGACCAGCGACGCCCCCGAGTATAAGCCTTGGGCTCTCGTGATTCAAG
ACTCGAATGGCGAGAACAAGATCAAGATGCTCAGCGGTGGCAGCGGCGGTTCGGGTGGTTCGACCAA
CCTCAGCGACATCATCGAAAAGGAGACCGGCAAGCAGCTCGTCATCCAAGAGAGCATTCTCATGCTGC
CCGAGGAGGTGGAGGAGGTCATTGGCAACAAGCCCGAGAGCGACATTCTCGTGCACACCGCCTACGA
TGAGAGCACCGACGAGAACGTGATGCTGCTGACCAGCGATGCCCCCGAGTACAAGCCTTGGGCTCTG
GTCATTCAAGACAGCAACGGCGAGAACAAAATCAAGATGCTGAGCGGCGGTAGCGACCCCAAGAAG
AAACGCAAGGTTGATCCGAAGAAGAAGCGGAAGGTTCCTCCGAGGAAACGTGCCAAAACAGAAGAT
GAGGGAAGCGGAGCTACTAACTTCAGCCTGCTGAAGCAGGCTGGAGACGTGGAGGAGAACCCTGGA
CCTATGAGCCCAGAACGACGCCCGGCCGACATCCGCCGTGCCACCGAGGCGGACATGCCGGCGGTCT
GCACCATCGTCAACCACTACATCGAGACAAGCACGGTCAACTTCCGTACCGAGCCGCAGGAACCGCA
GGAGTGGACGGACGACCTCGTCCGTCTGCGGGAGCGCTATCCCTGGCTCGTCGCCGAGGTGGACGGC
GAGGTCGCCGGCATCGCCTACGCGGGCCCCTGGAAGGCACGCAACGCCTACGACTGGACGGCCGAG
TCGACCGTGTACGTCTCCCCCCGCCACCAGCGGACGGGACTGGGCTCCACGCTCTACACCCACCTGCT
GAAGTCCCTGGAGGCACAGGGCTTCAAGAGCGTGGTCGCTGTCATCGGGCTGCCCAACGACCCGAGC
GTGCGCATGCACGAGGCGCTCGGATATGCCCCCCGCGGCATGCTGCGGGCGGCCGGCTTCAAGCACG
GGAACTGGCATGACGTGGGTTTCTGGCAGCTGGACTTCAGCCTGCCGGTACCGCCCCGTCCGGTCCT
GCCCGTCACCGAGATTTGATGAACTTAACGTTACTGAAATCATCAAACAGCTTGACGAATCTGGATATA
AGATCGTTGGTGTCGATGTCAGCTCCGGAGTTGAGACAAATGGTGTTCAGGATCTCGATAAGATACGTT
CATTTGTCCAAGCAGCAAAGAGTGCCTTCTAGTGATTTAATAGCTCCATGTCAACAAGAATAAAACGC
GTTTTCGGGTTTACCTCTTCCAGATACAGCTCATCTGCAATGCATTAATGCATTGACTGCAACCTAGTAA
CGCCTTTCAGGCTCCGGCGAAGAGAAGAATAGCTTAGCAGAGCTATTTTCATTTTCGGGAGACGAGAT
CAAGCAGATCAACGGTCGTCAAGAGACCTACGAGACTGAGGAATCCGCTCTTGGCTCCACGCGACTAT
ATATTTGTCTCTAATTGTACTTTGACATGCTCCTCTTCTTTACTCTGATAGCTTGACTATGAAAATTCCGT
CACCAGCTCCTGGGTTCGCAAAGATAATTGCATGTTTCTTCCTTGAACTCTCAAGCCTACAGGACACAC

ATTCATCGTAGGTATAAACCTCGAAATCATTTCCTACTAAGATGGTATACAATAGTAACCATGCATGGTT
GCCTAGTGAATGCTCCGTAACACCCAATACGCCGGCCGAAACTTTTTTACAACTCTCCTATGAGTCGTT
TACCCAGAATGCACAGGTACACTTGTTTAGAGGTAATCCTTCTT

evoCDA1max

>Ptef1-NLS-AID-xnCas9-3.7-linker-UGI-P2A-Bar-TtrpC

TCTGGCCATGTTCCGCATCTATCTACATCAGTTCAGTCTTTGCCCTGTTGGTCTTGCTAGCGGCGATGC
TATGAAAATCTATCAATTAATGGTGTCCTTTTTCGATGCGAGTACACTACTCATTTCGTTTCCGAATCG
CCATCTCTTCGACCATTGCTGAACTAATTCAATCTTAGGTAGTCTGCTGTTCCTTGTTTTCCATTTCCTT
GTCTTAAGTAAAACCAACTGGCACACCTCGAAACACGCTTGACGGATGGACAGTAGAATTGACCGTG
TACGTACATGTACCTTGACGTCCTCCGAGGTTCGACATCAGGGTTCGTCATAGGGAGTGAAACACCC
GCCATGATTCCGTAGCCGCGCGCGAAGATACGAAGCAGATATTTCACGGACATGGCGGAGATACTTG
TTTCCCGTACTAAGGTAGTCATGTCGGAGACATCTGAACGACAGAGCTGGCCAAGAGAACCGACCAG
TTGCCCCAGGACGATCTAGACAAAAAAAAAGAGAGATGAGTGGGCCACTTTTGCCACAACATCGAC
GGCCCTGCGACCGCCCCCAGGCAAACAAACAAACCGCCGAACAATAATACTTTTGTCATTTTAGGAG
GAGCGTTGTATGGATAAAAACAACATCTCGTTGCTGCAGAATGTGGACTTCAAACTTGCAGAAAATG
GGAGGCGGATTTGCATGATCGGAGGGTAGTTGACTCACGCCGCAGGCTGCAAATCCGTCCTCCATTA
TTCCATGAACAACTTCGTAAGGTTGGGCTGAGCGCCAATGCCTAACGGACCGGGGGCCACAGCGCAA
CGTCCCACTTAAAGGCCAGCGTGACATGCCAGTTCCATACCAAGTAGTGGCACCAGAGGCGGCCAAT
GCTCAGTAAGGGCAGGGAGGGAGGCTCAAACGATTGGCAAAAAGAGGGGCTTGCCAGTTCAGTTCC
CTGTGCGAGCGCGAGAGGGGCAGTTTCAAATCTGGAGGGGTGTGTTGCGCTGGTCTGAAGAGAAAG
AGAAGACTGTACTTAATAATTGTTCAAAGAGTCCATCATCGCGTTGCGGACTCCTCTAGCTGTATTTA
GAGCCCTATCATTACTTGTCGGGTGCGAATCAAAATACCGGGATGCAGCCCTCTGGCGATTTGCATG
CGGTTGTGGAGGAAGTGAAGCCTGAATCGCGGGGCTGGGCGGCAAAGCACGACGTGAAATTCCTGG
CGAAATTCGAGGGCTTGCCCCACCGTGGTTGAAGTTTTTGTGCTGCGTAACCCCACCAACCCGCCTTG
CCCCTCCCGCCTGCCCATAAAAACTTCGACCCCTCCTCAAATCTTCTTCGATTCTTCCTCTTCACTTCC
TTCGTCGGCATACCTGATTCAAGCAATCACCTGCCACTTTCAAGTGCGTATACCATCATCGATACACT
GGTTCTTGACAAGTACATCGTCTCTAACTTTCCTTTTTGCAGTTTTCATTAAGCGCAAGTCGCCAGTTT
CGTTCTTCAGAATGCCTCCAAGGAAACGCGCGAAGACCGAGGACGAGAAGCGCACCGCCGACGGCT
CCGAGTTCGAGTCCCCTAAGAAAAAGCGCAAGGTGACCGACGCCGAGTACGTCCGCATCCACGAGAA
GCTCGATATCTACACCTTCAAGAAGCAGTTCTCCAACAACAAGAAGTCCGTCAGCCACCGCTGCTACG
TCCTCTTCGAGCTGAAGCGCCGTGGCGAGCGCCGTGCCTGCTTCTGGGGCTACGCCGTCAACAAGCCT
CAGTCCGGCACCGAGCGCGGCATCCATGCCGAGATCTTCTCCATCCGCAAGGTCGAGGAATACCTCCG
CGATAACCCTGGCCAGTTCACTATCAACTGGTACTCCAGCTGGTCCCCATGCGCCGATTGCGCCGAGA
AGATCCTCGAGTGGTACAATCAAGAGCTGCGCGGCAACGGTCATACCCTCAAGATCTGGGTCTGCAAG
CTCTACTACGAGAAGAACGCCCGCAACCAGATCGGCCTCTGGAACCTGCGCGATAATGGCGTCGGCCT
CAACGTCATGGTCAGCGAGCATTACCAGTGCTGCCGCAAGATCTTCATCCAGTCCAGCCATAACCAGC
TCAACGAGAACCGCTGGCTCGAAAAGACCCTCAAGCGCGCTGAGAAGCGCCGCTCCGAGCTGTCCAT
CATGTTCCAGGTCAAGATCCTCCATACCACCAAGTCTCCCGCCGTCAGCGGCGGCTCCTCCGGCGGCA
GCTCCGGCTCCGAGACTCCTGGCACCTCCGAGAGCGCTACCCCTGAGTCCTCCGGTGGCTCCAGCGG
CGGTTCCGATAAGAAGTACTCCATCGGCCTCGCCATCGGCACCAACTCCGTCGGCTGGGCCGTCATC
ACCGATGAGTACAAGGTCCCTTCCAAGAAGTTCAAGGTCCTCGGCAACACCGATCGCCATTCCATCA
AGAAGAACCTGATCGGCGCCCTCCTGTTCGATTCCGGCGAAACCGCCGAGGCCACCCGCCTTAAACG
CACCGCCCGTCGCCGCTACACCCGCCGCAAGAACCGCATCTGCTACCTCCAAGAAATCTTCTCCAAC

GAGATGGCCAAGGTCGATGATAGCTTCTTCCACCGCCTCGAAGAGTCCTTCCTGGTCGAAGAGGATA
AGAAGCACGAGCGCCATCCTATCTTCGGCAACATCGTCGATGAGGTCGCCTACCATGAGAAGTACCC
TACCATCTACCATCTCCGCAAGAAGCTCGTCGATTCCACCGATAAGGCCGATCTCCGCCTCATCTACC
TCGCCCTCGCCCATATGATCAAGTTCCGCGGCCATTTCCTCATCGAGGGCGATCTCAACCCTGATAAC
TCCGATGTCGATAAGCTGTTCATCCAGCTCGTCCAGACCTACAACCAGCTGTTCGAGGAAAACCCTAT
CAACGCCTCCGGCGTCGATGCCAAGGCCATCCTCTCCGCTCGCCTCTCCAAGTCTCGCCGCCTTGAGA
ACCTTATCGCCCAGCTCCCTGGCGAGAAGAAGAACGGCCTCTTCGGCAACCTGATCGCCCTCTCCCTC
GGCCTCACCCCTAACTTCAAGTCCAACTTCGATCTCGCCGAGGATACCAAGCTCCAGCTCTCCAAGG
ATACCTACGATGATGATCTCGATAACCTCCTCGCCCAGATCGGCGATCAGTACGCCGATCTGTTCCTC
GCCGCCAAGAACCTCTCCGATGCCATCCTCCTCTCCGACATCCTCCGCGTCAACACCGAGATCACCAA
GGCCCCTCTGTCCGCCTCCATGATCAAGCTCTACGATGAGCATCATCAGGACCTCACCCTGCTCAAGG
CCCTCGTCCGCCAGCAGCTCCCTGAGAAGTACAAAGAGATTTTCTTCGATCAGTCCAAGAACGGCTA
CGCCGGCTACATCGATGGCGGCGCTTCCCAAGAAGAGTTCTACAAGTTCATCAAGCCTATCCTTGAG
AAGATGGATGGCACCGAGGAACTCCTCGTCAAGCTCAACCGCGAGGACCTCCTCCGCAAGCAGCGC
ACCTTCGATAACGGCATCATCCCTCATCAAATCCATCTCGGCGAGCTGCATGCCATCTTGCGCCGCCA
AGAGGATTTCTACCCATTCCTCAAGGATAACCGCGAGAAGATCGAAAAGATTCTCACCTTCCGCATC
CCTTACTACGTCGGCCCTCTCGCTCGCGGCAACTCCCGCTTCGCCTGGATGACCCGCAAGTCCGAGGA
AACCATCACCCCTTGGAACTTCGAGAAAGTCGTCGATAAGGGCGCCTCCGCCCAGTCCTTCATCGAG
CGCATGACCAACTTCGATAAGAACCTCCCTAACGAGAAGGTCCTCCCTAAGCACTCCCTGCTCTACG
AGTACTTCACCGTCTACAACGAGCTGACCAAGGTCAAGTACGTCACCGAGGGTATGCGCAAGCCTGC
CTTCCTGTCCGGCGATCAGAAGAAGGCCATCGTCGATCTGCTGTTCAAGACCAACCGCAAGGTCACC
GTCAAGCAGCTCAAAGAGGATTACTTCAAGAAAATCGAGTGCTTCGATTCCGTCGAGATCAGCGGCG
TCGAGGACCGCTTCAACGCCTCCCTCGGAACCTACCATGATCTCCTCAAGATTATCAAGGATAAGGA
TTTCCTCGACAACGAGGAAAACGAGGACATCCTTGAGGACATCGTCCTCACCCTCACCCTCTTCGAG
GACCGCGAAATGATCGAGGAACGCCTCAAGACCTACGCCCATCTCTTCGATGATAAGGTCATGAAGC
AGCTCAAGCGCCGTCGCTACACCGGCTGGGGTCGCCTCTCCCGCAAGCTCATCAACGGCATCCGCGA
TAAGCAGTCCGGCAAGACTATCCTCGATTTCCTCAAGTCCGATGGCTTCGCCAACCGCAACTTCATTC
AGCTCATCCATGATGATTCCCTCACCTTCAAAGAGGACATCCAGAAGGCCCAGGTCAGCGGCCAGGG
CGATTCCCTCCATGAGCATATCGCCAACCTCGCCGGCTCCCCTGCCATCAAGAAGGGCATCCTCCAG
ACCGTCAAGGTCGTCGATGAGCTGGTCAAGGTCATGGGCCGCCATAAGCCTGAGAACATCGTCATCG
AGATGGCCCGCGAGAACCAGACCACCCAGAAGGGCCAGAAGAACTCCCGCGAGCGCATGAAGCGCA
TCGAGGAAGGCATCAAAGAGCTGGGCAGCCAAATCCTCAAAGAGCATCCTGTCGAGAACACCCAGC
TCCAGAACGAGAAGCTCTACCTCTACTACCTCCAGAACGGCCGCGATATGTACGTCGATCAAGAGCT
GGACATCAACCGCCTCTCCGATTACGATGTCGATCATATCGTCCCTCAGTCCTTCCTGAAGGATGATT
CCATCGATAACAAGGTCCTCACCCGCTCCGATAAGAACCGCGGCAAGTCCGATAACGTCCCTTCCGA
AGAGGTCGTCAAGAAGATGAAGAACTACTGGCGCCAGCTCCTCAACGCCAAGCTCATCACCCAGCGC
AAGTTCGATAACCTCACCAAGGCCGAGCGCGGTGGCCTCTCCGAGCTGGATAAGGCCGGCTTCATCA
AGCGCCAGCTCGTCGAAACCCGCCAGATCACCAAGCACGTCGCCCAAATCCTCGATTCCCGCATGAA
CACCAAGTACGATGAGAACGATAAGCTCATCCGCGAAGTCAAGGTCATCACCCTCAAGTCCAAGCTC
GTCAGCGATTTCCGCAAGGATTTCCAGTTCTACAAGGTCCGCGAGATCAACAACTACCATCATGCCC
ATGATGCCTACCTCAACGCCGTCGTCGGCACCGCCCTCATCAAGAAGTACCCCAAGCTCGAATCCGA
GTTCGTCTACGGTGATTACAAGGTCTACGATGTCCGCAAGATGATCGCCAAGTCCGAGCAAGAGATC
GGCAAGGCTACCGCCAAGTACTTCTTCTACTCCAACATCATGAATTTCTTCAAGACCGAAATCACCCT
CGCCAACGGCGAAATCCGCAAGCGCCCTCTCATCGAGACTAACGGCGAGACTGGCGAGATCGTCTGG

GATAAGGGCCGCGATTTCGCCACCGTCCGCAAGGTCCTCTCCATGCCTCAGGTCAACATCGTCAAGA
AAACCGAGGTCCAGACCGGCGGCTTCTCCAAAGAGTCCATCCTCCCCAAGCGCAACTCCGATAAGCT
GATCGCCCGCAAGAAGGATTGGGACCCTAAGAAGTACGGCGGCTTCGATTCCCCTACCGTCGCCTAC
TCCGTCCTCGTCGTCGCCAAGGTCGAGAAGGGCAAGTCCAAGAAGCTCAAGTCCGTCAAAGAGCTGC
TCGGCATCACTATTATGGAACGCTCCAGCTTCGAGAAGAACCCTATCGATTTCCTTGAGGCCAAGGG
CTACAAAGAGGTCAAGAAGGACCTCATCATCAAGCTCCCCAAGTACTCCCTGTTCGAGCTTGAGAAC
GGCCGCAAGCGCATGCTCGCCTCCGCCGGTGTGCTTCAGAAGGGCAACGAGCTGGCCCTGCCTTCCA
AGTACGTCAACTTCCTCTACCTCGCCTCCCATTACGAGAAGCTCAAGGGCTCCCCTGAGGATAACGA
GCAGAAGCAGCTGTTCGTCGAGCAGCATAAGCACTACCTCGATGAGATCATCGAGCAGATCAGCGA
GTTCTCCAAGCGCGTCATCCTCGCCGATGCCAACCTCGATAAGGTCCTGTCCGCCTACAACAAGCACC
GCGATAAGCCTATCCGCGAGCAGGCCGAGAACATCATCCATCTCTTCACCCTCACCAACCTCGGTGC
CCCTGCCGCCTTCAAGTACTTCGATACCACCATCGATCGCAAGCGCTACACCTCCACCAAAGAGGTC
CTGGACGCCACCCTCATCCATCAGTCCATCACCGGCCTCTACGAAACCCGCATCGATCTCTCCCAGCT
CGGCGGCGACTCGGGCGGTAGCGGTGGTTCGGGCGGCAGCACGAACCTCAGCGACATTATCGAGAAG
GAGACGGGCAAGCAGCTCGTCATCCAAGAGTCGATCCTCATGCTGCCCGAGGAGGTCGAAGAGGTCA
TCGGCAACAAGCCCGAGTCGGACATCCTCGTCCATACCGCCTACGACGAGAGCACCGATGAAAACGT
CATGCTGCTGACCAGCGACGCCCCCGAGTATAAGCCTTGGGCTCTCGTGATTCAAGACTCGAATGGCG
AGAACAAGATCAAGATGCTCAGCGGTGGCAGCGGCGGTTCGGGTGGTTCGACCAACCTCAGCGACAT
CATCGAAAAGGAGACCGGCAAGCAGCTCGTCATCCAAGAGAGCATTCTCATGCTGCCCGAGGAGGTG
GAGGAGGTCATTGGCAACAAGCCCGAGAGCGACATTCTCGTGCACACCGCCTACGATGAGAGCACCG
ACGAGAACGTGATGCTGCTGACCAGCGATGCCCCCGAGTACAAGCCTTGGGCTCTGGTCATTCAAGAC
AGCAACGGCGAGAACAAAATCAAGATGCTGAGCGGCGGTAGCGACCCCAAGAAGAAACGCAAGGTT
GATCCGAAGAAGAAGCGGAAGGTTCCTCCGAGGAAACGTGCCAAAACAGAAGATGAGGGAAGCGGA
GCTACTAACTTCAGCCTGCTGAAGCAGGCTGGAGACGTGGAGGAGAACCCTGGACCTATGAGCCCAG
AACGACGCCCGGCCGACATCCGCCGTGCCACCGAGGCGGACATGCCGGCGGTCTGCACCATCGTCAA
CCACTACATCGAGACAAGCACGGTCAACTTCCGTACCGAGCCGCAGGAACCGCAGGAGTGGACGGA
CGACCTCGTCCGTCTGCGGGAGCGCTATCCCTGGCTCGTCGCCGAGGTGGACGGCGAGGTCGCCGGC
ATCGCCTACGCGGGCCCCTGGAAGGCACGCAACGCCTACGACTGGACGGCCGAGTCGACCGTGTACG
TCTCCCCCCGCCACCAGCGGACGGGACTGGGCTCCACGCTCTACACCCACCTGCTGAAGTCCCTGGA
GGCACAGGGCTTCAAGAGCGTGGTCGCTGTCATCGGGCTGCCCAACGACCCGAGCGTGCGCATGCAC
GAGGCGCTCGGATATGCCCCCCGCGGCATGCTGCGGGCGGCCGGCTTCAAGCACGGGAACTGGCATG
ACGTGGGTTTCTGGCAGCTGGACTTCAGCCTGCCGGTACCGCCCCGTCCGGTCCTGCCCGTCACCGAG
ATTTGATGAACTTAACGTTACTGAAATCATCAAACAGCTTGACGAATCTGGATATAAGATCGTTGGTGT
CGATGTCAGCTCCGGAGTTGAGACAAATGGTGTTCAGGATCTCGATAAGATACGTTCATTTGTCCAAGC
AGCAAAGAGTGCCTTCTAGTGATTTAATAGCTCCATGTCAACAAGAATAAAACGCGTTTTCGGGTTTAC
CTCTTCCAGATACAGCTCATCTGCAATGCATTAATGCATTGACTGCAACCTAGTAACGCCTTTCAGGCT
CCGGCGAAGAGAAGAATAGCTTAGCAGAGCTATTTTCATTTTCGGGAGACGAGATCAAGCAGATCAAC
GGTCGTCAAGAGACCTACGAGACTGAGGAATCCGCTCTTGGCTCCACGCGACTATATATTTGTCTCTAA
TTGTACTTTGACATGCTCCTCTTCTTTACTCTGATAGCTTGACTATGAAAATTCCGTCACCAGCTCCTGG
GTTCGCAAAGATAATTGCATGTTTCTTCCTTGAACTCTCAAGCCTACAGGACACACATTCATCGTAGGT
ATAAACCTCGAAATCATTTCCTACTAAGATGGTATACAATAGTAACCATGCATGGTTGCCTAGTGAATGC
TCCGTAACACCCAATACGCCGGCCGAAACTTTTTTACAACTCTCCTATGAGTCGTTTACCCAGAATGCA
CAGGTACACTTGTTTAGAGGTAATCCTTCTT

Note S2. Sequences of U6-sgRNA

>U6-esgRNA

AGGATCGGTGGAGTGAAGTTCGGAATCGAGGTTCGGCGATGGGTCGTAAGCATGGCGACTTCGAACT
TACTTGCACTGGCAAGCGTTGCCAGAACGGCGAGAAAAAGAAGGGTAAGCGATATTCGCGTCATGAT
GGACTGTTCCTTTTGGAACAGTAGTTGTTGTGGGAAGACTATGTCACACTTGCCCACCTGCAAGGCCA
GGGTCGTGGTCGAACGAGACCAGCCTCGGCGCTGCTGGGAGCTCAAGATGGGCACGTTTGATTCGTT
AGACGTCAACAAGGCTGGAGTTCCTAGTGACAGCCAAAGGCACAGCCACATTAAGTGGCGCTTTATCT
GTCCACTAAGGTTCAATTGTGGCTTTGAGCCGCGCAGTGTGCAGTCGTGCATTGGCCACCTAGCTAGC
AGTATTTAAGATCCTCTTCTCTCCCGAGATCTTCCTCCTCTTCTTTTCTTTCTTTCCTCNNNNNNNNNNN
NNNNNNNNNGTTTTAGAGCTATGCTGGAAACAGCATAGCAAGTTTAAATAAGGCTAGTCCGTTATCAA
CTTGAAAAAGTGGCACCGAGTCGGTGCTTTTTTT
